# Habitat types and megabenthos composition from three sponge-dominated high-Arctic seamounts

**DOI:** 10.1038/s41598-022-25240-z

**Published:** 2022-11-29

**Authors:** Tanja Stratmann, Erik Simon-Lledó, Teresa Maria Morganti, Anna de Kluijver, Andrey Vedenin, Autun Purser

**Affiliations:** 1 Department of Earth Sciences, Utrecht University, Vening Meineszgebouw A, Princetonlaan 8, 3584 CB Utrecht, The Netherlands; 2 HGF MPG Joint Research Group for Deep-Sea Ecology and Technology, Max Planck Institute for Marine Microbiology, Celsiusstraße 1, 28359 Bremen, Germany; 3 Department of Ocean Systems, NIOZ – Royal Netherlands Institute for Sea Research, Landsdiep 4, 1797 SZ ‘t Horntje (Texel), The Netherlands; 4 Ocean BioGeosciences, National Oceanography Centre, European Way, Southampton, SO14 3ZH UK; 5 Marine Chemistry Department, Leibniz Institute for Baltic Sea Research Warnemünde, Seestraße 15, 18119 Rostock, Germany; 6 Marine Biology Section, Senckenberg am Meer, Südstrand 40, 26382 Wilhelmshaven, Germany; 7 Alfred Wegener Institute, Helmholtz Centre for Polar and Marine Research, Am Handelshafen 12, 27570 Bremerhaven, Germany

**Keywords:** Zoology, Ecology, Ocean sciences

## Abstract

Seamounts are isolated underwater mountains stretching > 1000 m above the seafloor. They are identified as biodiversity hotspots of marine life, and host benthic assemblages that may vary on regional (among seamounts) and local (within seamounts) scales. Here, we collected seafloor imagery of three seamounts at the Langseth Ridge in the central Arctic Ocean to assess habitats and megabenthos community composition at the Central Mount (CM), the Karasik Seamount (KS), and the Northern Mount (NM). The majority of seafloor across these seamounts comprised bare rock, covered with a mixed layer of sponge spicule mats intermixed with detrital debris composed of polychaete tubes, and sand, gravel, and/or rocks. The megabenthos assemblages consisted of in total 15 invertebrate epibenthos taxa and 4 fish taxa, contributing to mean megabenthos densities of 55,745 ind. ha^−1^ at CM, 110,442 ind. ha^−1^ at KS, and 65,849 ind. ha^−1^ at NM. The faunal assemblages at all three seamounts were dominated by habitat-forming Tetractinellida sponges that contributed between 66% (KS) and 85% (CM) to all megabenthos. Interestingly, taxa richness did not differ at regional and local scale, whereas the megabenthos community composition did. Abiotic and biogenic factors shaping distinct habitat types played a major role in structuring of benthic communities in high-Arctic seamounts.

## Introduction

Seamounts are isolated subaquatic mountains of (mostly) volcanic origin that rise at least 1000 m above the surrounding seafloor^1^. With a global abundance of ~ 10,000^2^ to ~ 125,000^3^ seamounts, they cover a minimum ~ 8,000,000 km^2^^2^ and form one of the largest biomes on our planet^4^. Seamounts are often hotspots of deep-water biodiversity^5–8^ and can support higher species abundances than surrounding continental margins and continental slopes^9^. This phenomenon is known as the ‘seamount oasis hypothesis’^9^ that asserts that benthic invertebrates occur in higher densities and biomasses on seamounts than in other habitats in the deep sea^9^. Seamounts can also influence the overlying water column and affect the microbial community^10,11^, phytoplankton^12^, zooplankton^13^, and ultimately large fish^1^, which is known as the ‘seamount effect’^13^.

Regional variations in benthic assemblages among seamounts can be driven by differences in latitude^14–16^, longitude^17^, food supply^18^, water depth^15,16,19–21^, or distance from shore^22^. Structuring factors can also be region-specific, for instance Boschen et al.^23^ identified magnetivity (i.e., a proxy for hydrothermal activity^24^) as main driver of differences in the benthic composition among three seamounts in New Zealand. Overall, many factors known to drive biological community variability in the deep sea are related with water depth (i.e., temperature, pressure, oxygen concentration, or food-availability), which restricts most benthic fauna to a limited bathymetric range^25^. As a result, available habitat for a given population or community can be fragmented across the seamounts (and continental slopes, e.g.,^26^) within a region^27^, particularly in areas with high variability in water depth at summits. In addition, most deep-sea benthic fauna are thought to exhibit a biphasic life cycle between the release of pelagic planktotrophic or lecithotrophic larvae (i.e., respectively plankton-, or yolk-feeding, e.g.,^28^) to the water column for dispersal and subsequent settlement on the substrate for benthic/sessile adult stage. As such, connectivity between species populations on different seamounts is thought to be largely controlled by regional factors affecting larvae transport, development, or resilience, such as food availability and temperature within the water column, hydrographic retention mechanisms, or the presence of suitable habitat where propagules ultimately settle^27^. However, regional gradients and larval dispersal dynamics can be strongly modulated by small-scale processes across seamounts^23,27,29,30^. Consequently, integration of local to regional observational scales is often essential to accurately assess spatial patterns in seamount communities.

Variations in benthic assemblages within seamounts have been related to changes in seabed composition (e.g., hard substrate availability)^16,19,23,31^, slope^16,32^, currents^32,33^, or food supply^32^; all factors that typically covary with water depth^19,23,33,34^. As a result, community composition tends to be depth-stratified within seamounts^6,27^. However, essential niche requirements, such as a minimum rate of food supply^35^ or the presence of hard substrate^36,37^ for many sessile taxa, can be strongly modulated by the topographical complexity of a seamount in interaction with the surrounding water masses^38^. Similarly, aggregations of framework-building fauna can generate a habitat for other species, such as is the case with cold-water coral reefs^35,39^ and sponge grounds^40,41^. Acting as ecosystem engineers, they enhance local habitat heterogeneity rates and thereby increase alpha diversity rates^42^. Given this wealth of possible drivers operating locally, and cumulative effects from their interactions, benthic assemblages within a seamount tend to exhibit rapid shifts in composition across space^43^, usually reflecting the ranges of one or more environmental gradients that define the boundaries of different habitat types.

There are likely more than 300 seamounts beneath Arctic waters^44^, but only a few have been the subjects of ecological investigations. Sponge grounds (i.e., sites where these sponges reach densities of 0.5–1 m^−2^ (still image surveys^45^) to 0.03–0.1 m^−2^ (video surveys^40^)) appear to be one of the most commonly found seamount habitats in high latitudes, as they have also been observed on N Atlantic seamounts (e.g., 40°–75° N latitude belt;^46,47^). Roberts et al.^48^ suggested that short-timescale environmental variability combined with the generally nutrient rich sub-surface water masses generated at the interface of intermediate and deep-sea water masses^49^ might potentially enhance the development of sponge aggregations and other megafauna (e.g., ascidians, cnidarians, echinoderms, and demersal fish) at the Schulz Bank seamount (73.5° N) on the Arctic Mid-Ocean Ridge. However, very little is known about the diversity of benthic communities in seamounts at higher Arctic latitudes (e.g., > 75° N; see also^50^). Hence, it remains unclear what the key factors structuring Arctic seamount communities across different spatial scales are, e.g., do these differ from those shaping lower latitude seamount communities?

The sponge order Tetractinellida includes deep-sea species that occur in cold water masses of the Arctic and North Atlantic^49^. Their distribution ranges from seamounts in the central Arctic Ocean^50,51^ and Nordic Seas^52,53^ to Norwegian fjords^54–56^, the Mid-Atlantic Ridge^46^, along the continental shelves of Europe, Canada and the US^40,47,57–59^, around Iceland and the Faroe Islands^60,61^ to New England seamounts in the central North Atlantic^62^. Sponge grounds usually exhibit an increased associated benthic diversity and biomass when compared with adjacent non-sponge habitats^41^. Sponges typically enhance the complexity of habitats by increasing the (three-dimensional) hard surface area available for other fauna to interact (e.g., settle, reproduce, and/ or hide^37,63–66^). For instance, at the Schulz Bank seamount large Tetractinellida sponges like *Geodia* sp. and *Stelletta* sp. typically have ascidians or other sponges (such as the encrusting sponge *Hexadella dedritifera*) growing along their edges^67^. Such dense sponge community act not only as ecosystem engineers, but they also play an important role in nutrient and matter cycling. By filtering large volume of water (up to 2000 L m^−2^ d^−1^ in case of *Geodia* sp.^40^), they efficiently retain pico-nanoplankton cells and process dissolved compounds acting as sink and/or source of nutrients and organic matter.

**Figure 1:**
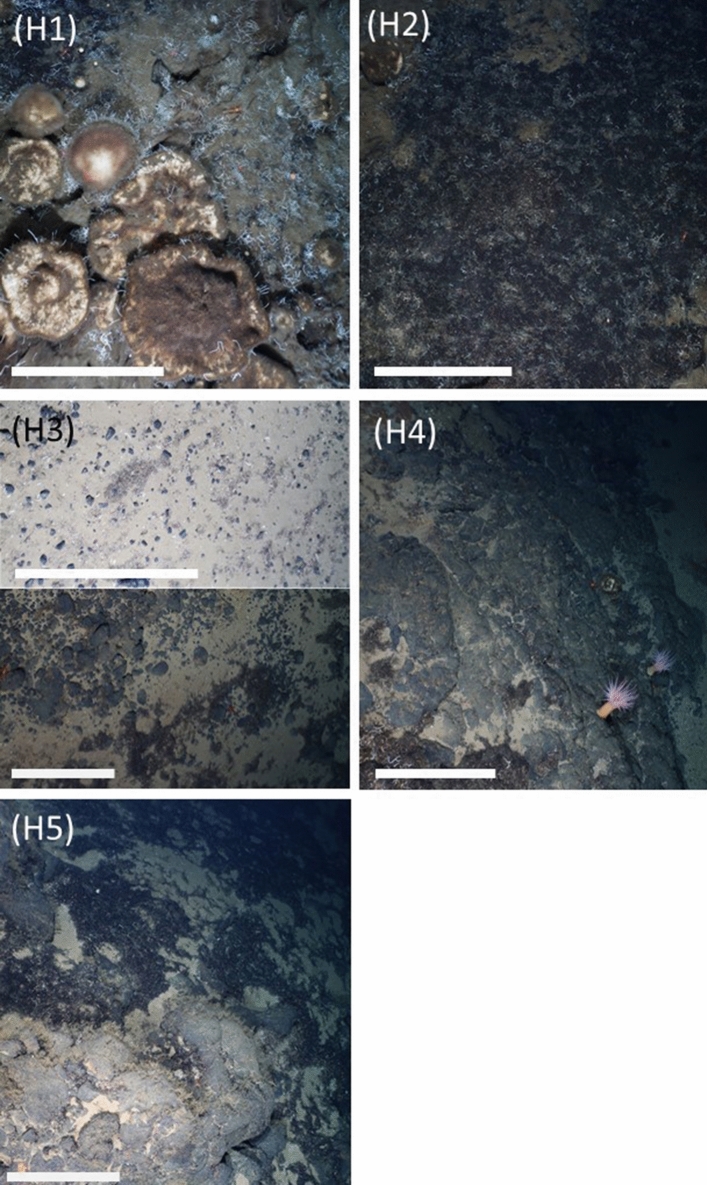
Habitat types identified over the three seamounts: (H1) dense sponge grounds of Tetractinellida gen. indet. sponges extending on top of sponge-produced spicule mats (partly comparable to habitat category d in^50^), (H2) mats of Serpulidae indet. and Siboglinidae indet. tubes intermixed with sponge spicules covered with sulfide precipitates (includes habitat category b in^50^), (H3) sediment with gravel, (H4) bare rock, and (H5) mixed substrate (equivalent to habitat type c in^50^). The white bar represents 50 cm.

In this study, we used seabed imagery to investigate regional (between seamounts) and local (within seamounts) variations in benthic megafauna communities across the Langseth Ridge in the Central Arctic (86.6°–86.9° N; approximate water depth 600–2000 m). The Langseth Ridge is a chain of three seamounts (Central Mount, Karasik Seamount, and Northern Mount), which summits are dominated by dense aggregations of mobile sponges (a striking and previously unforeseen trait;^51^)^50^. We hypothesized that seabed composition (Fig. 1, five potential habitats: H1, dense sponge grounds; H2, mats of extensive polychaeta tubes and sponge spicules covered with sulfide precipitates; H3, sediment with gravel; H4, bare rock; and H5, mixed or undominated substrate) plays a large role in structuring megabenthic assemblages, i.e. delineating abrupt seabed community variations within arctic seamounts. In addition, we describe and discuss how common processes triggered by decaying sponges, e.g. “the sponge loop”^68–70^, might enhance local and regional habitat variations.

## Results

### Micro- and macrohabitat types

Most of the seafloor at the Central Mount (CM) was covered with bare rock (habitat type H4, 56% seabed coverage; Table S1, Fig. 1) and mixed substrate (H5, 30.9% seabed coverage; Table S1, Fig. 1), i.e., a mixed assemblage of sponge grounds with spicule mats, mats of polychaete tubes, sand with gravel, and/ or bare rocks. Habitat type H2, i.e., mats of Serpulidae gen. indet. and Siboglinidae gen. indet. tubes intermixed with sponge spicules and covered with sulfide precipitates, was not observed. The Karasik Seamount (KS) was dominantly covered by H4 (73.4%; Table S1) and the Northern Mount’s (NM) seabed consisted to 56.7% of habitat type H5 and to 30.6% of habitat type H4 (Table S1).

### Quantitative assessment

#### Variations in faunal density

**Figure 2:**
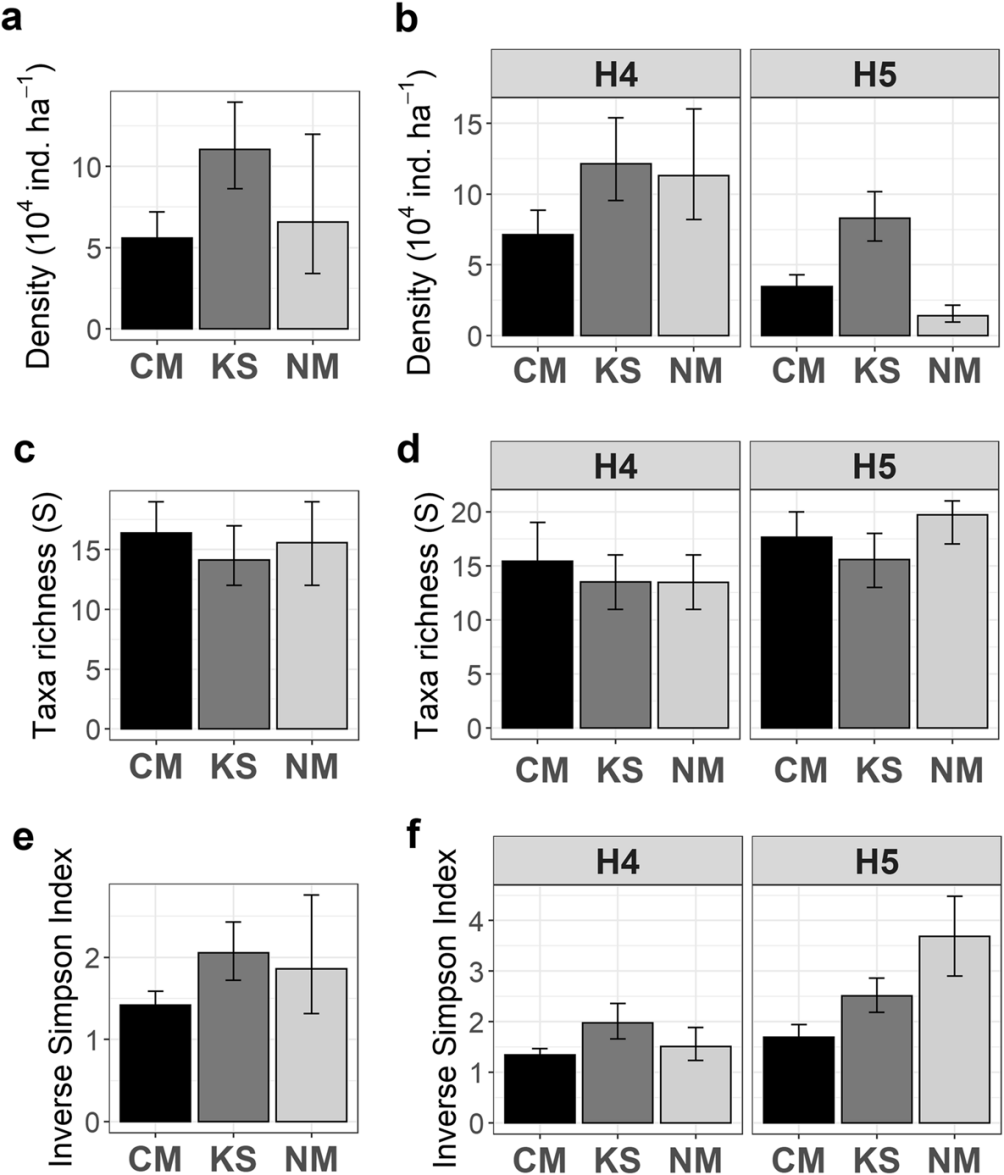
Regional variations in (**a**) megabenthos density (ind. ha^−1^), (**c**) taxa richness *S* (in ca. 1,000 specimens), (**e**) 1/Simpson index *D* (in ca. 1000 specimens), and local variations in (**b**) megabenthos density (ind. ha^−1^), (**d**) taxa richness *S* (in ca. 1000 specimens), and (**f**) 1/Simpson index *D* (in ca. 1,000 specimens) across the three Arctic seamounts (*CM* Central Mount, *KS* Karasik Seamount, *NM* Northern Mount) investigated. Bars indicate mean values across bootstrap-like sample sets surveyed in each study area (**a**, **c**, **e**) and in the predominant habitat types (H4 and H5) within each study area (**b**, **d**, **f**). Error bars represent CI 95%.

Megabenthos density exhibited substantial variations across the different areas investigated, both at the regional (between seamounts) and at the local (between habitats) scales. Mean faunal density at KS (mean density: 110,442 ind. ha^−1^; CI 95%: 86,253 − 139,541 ind. ha^−1^) was substantially larger than at CM (mean density: 55,745 ind. ha^−1^; CI 95%: 43,305–71,984 ind. ha^−1^) (Fig. 2a), whereas the assemblages in NM exhibited a large variability, ranging from 34,162 to 119,762 ind. ha^−1^ (in ca. 1000 specimen samples). Local assessments revealed that the high variability observed at NM was predominantly caused by large differences in faunal density between H4 (mean density: 113,118 ind. ha^−1^) and H5 (mean density: 14,123 ind. ha^−1^) (Fig. 2b). Densities were consistently smaller in H5 compared to H4 areas, but substantially different densities were also found between habitats of the same type across different seamounts (Fig. 2b), suggesting the existence of faunal density drivers operating at both local and regional scales. Taxon-specific densities specifically for H4 and H5 are presented in Table S2.

#### Variations in diversity

No substantial variations in taxa richness were observed across the different areas investigated, neither regionally nor locally (Fig. 2c–d). In contrast, heterogeneity diversity (i.e., 1/*D*, an index more sensitive to taxa evenness) was substantially higher at KS than at CM, whereas the assemblages at NM exhibited a large variability for this parameter (Fig. 2e). Similar to faunal density, local assessments revealed that the high variability observed at NM was predominantly caused by large differences in heterogeneity diversity between H4 (mean 1/*D*: 1.5 effective taxa) and H5 (mean 1/*D*: 3.7 effective taxa) (Fig. 2f). In turn, no major differences were observed in heterogeneity diversity between H4 and H5 areas at CM nor at KS (Fig. 2f). However, heterogeneity diversity in both H4 and H5 areas from KS were consistently higher than in respective H4 and H5 areas from CM, again suggesting the existence of diversity drivers operating at both local and regional scales.

#### Variations in assemblage composition

A total of 15 invertebrate epibenthos taxa and 4 fish taxa were identified in the image set across the three seamounts studied (Fig. S1). At KS 15 invertebrate and 3 fish taxa were observed, whereas 14 invertebrates and 4 fish taxa were detected at CM. At NM all taxa observed on the other seamounts were also found (Table S3).

**Figure 3:**
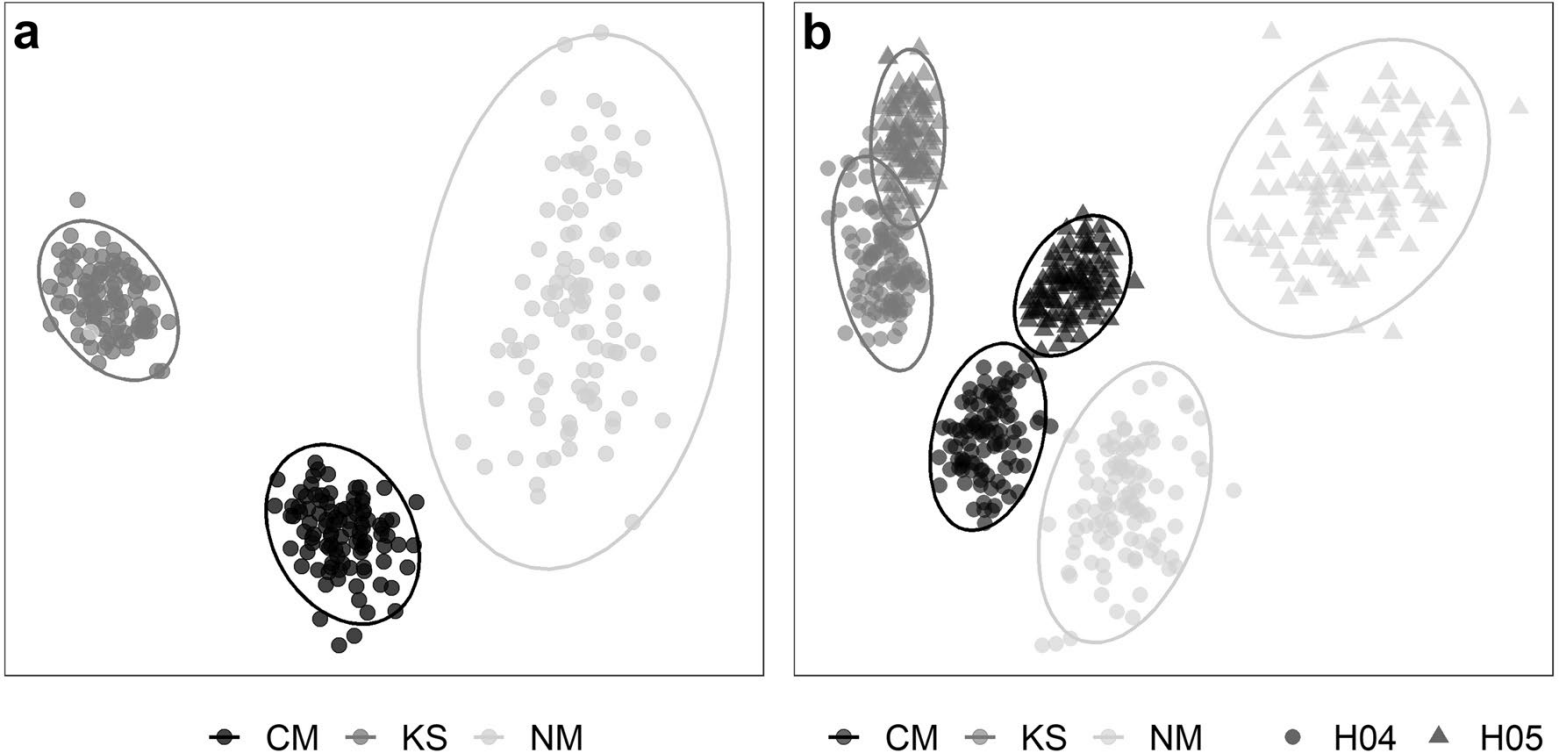
nMDS plots showing regional (between seamounts) and local variations (between habitat types) in faunal assemblage composition. (**a**) Regional assessment based on 100 randomly selected bootstrap-like samples for each study area (seamounts: *CM* Central Mount, *KS* Karasik Seamount, and *NM* Northern Mount) (nMDS stress: 0.09). (**b**) Local assessment based on 100 randomly selected bootstrap-like samples for the two dominant habitat types (H4 = bare rock, H5 = mixed substrate) in each study area (nMDS stress: 0.12). Ellipses represent CI 95%.

Multivariate analyses showed substantial variations among the assemblages of different areas investigated, both at the regional (between seamounts) and at the local (between habitats) scales. Non-metric multidimensional scaling (nMDS) ordination of regional assemblage composition data readily distinguished the bootstrap-like samples from the three study areas, particularly those from KS and NM (Fig. 3a), as assemblage dissimilarity was higher between these two areas (β_BC_: 32.1%) than between CM and the other two seamounts (β_BC_: 24.6–25%). A much larger variation was found, however, within the assemblages of NM than within both CM and KS assemblages (Fig. 3a). Local assessments revealed that the high within-sample variability observed at NM was predominantly caused by a high dissimilarity between the assemblages in H5 and H4 (β_BC_: 31.3%; Fig. 3b). The latter exhibited a high resemblance with the assemblage from H4 areas at CM (β_BC_: 22.3%). In contrast, dissimilarity between the assemblages of H4 and H5 was less pronounced within CM (β_BC_: 17.3%), and almost inexistent within KS (β_BC_: 13.8%; Fig. 3b, overlapping confidence intervals), suggesting a stronger control of regional drivers at CM and KS areas, and a stronger control of local drivers at NM area.

### Faunal assemblage at different seamounts

#### Central Mount

**Figure 4:**
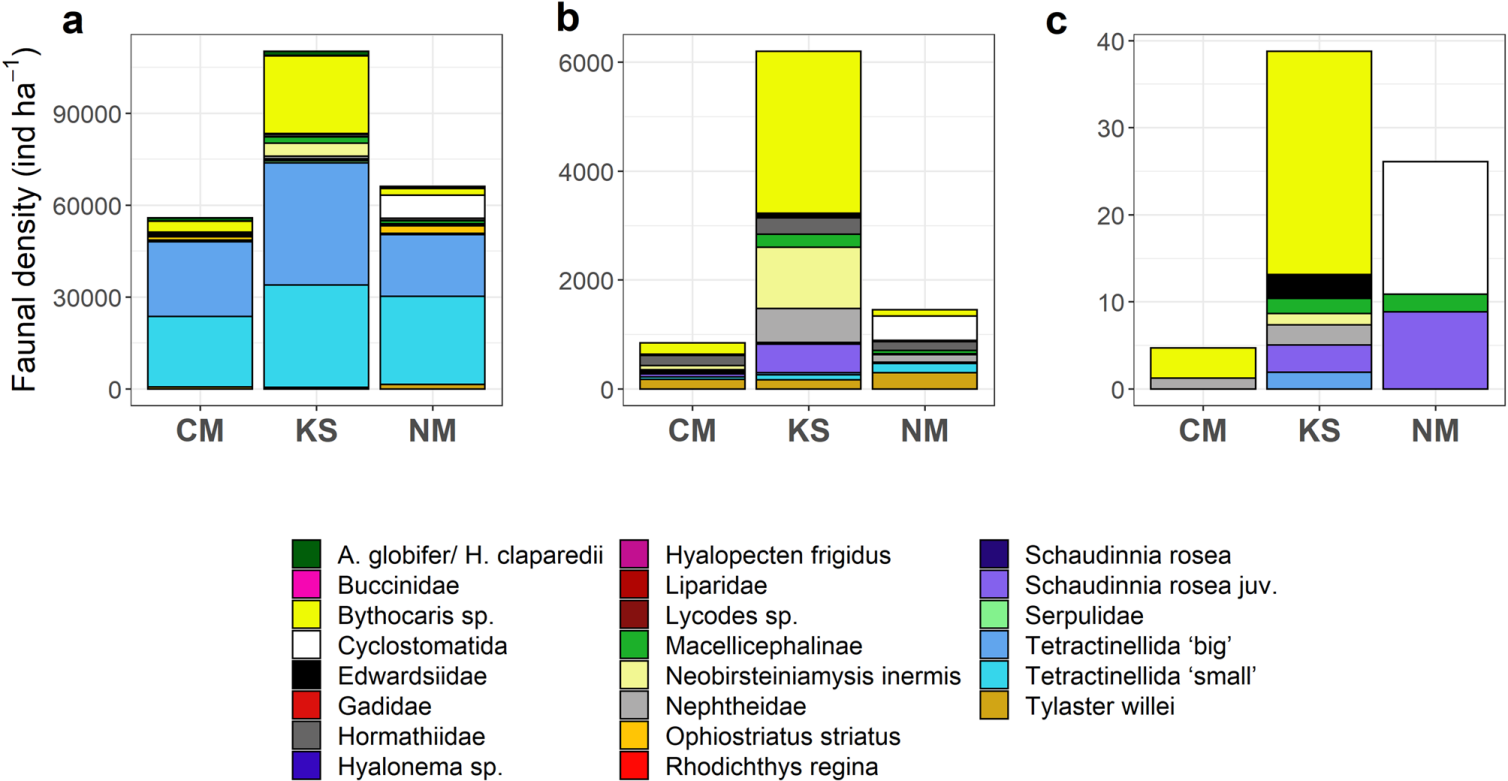
Invertebrate epibenthic megabenthos and fish densities at the Central Mount (CM), Karasik Seamount (KS), and Northern Mount (NM). (**a**) All fauna (ind. ha^−1^) observed at the different seamounts, (**b**) all fauna (ind. ha^−1^) that was observed being physically attached to Tetractinellida gen. indet. or walking/ crawling on top of Tetractinellida gen. indet., (**c**) all fauna (ind. ha^−1^) that was physically associated with Hexactinellida or was crawling on top of Hexactinellida.

The faunal assemblage at CM was clearly dominated by sponges of the order Tetractinellida gen. indet. (84.9% of all fauna; 23,070 ind. ha^−1^ sponges with < 8 cm diameter, 24,348 ind. ha^−1^ sponges with > 8 cm diameter; Fig. 4a, Table S3). The second, third, and fourth most abundant taxa were, respectively: the shrimp *Bythocaris* sp. indet. (3502 ind. ha^−1^; 6.27% of all fauna), the brittle star *Ophiostriatus striatus* sp. inc. (1133 ind. ha^−1^; 2.03% of all fauna), and the polychaetes *Apomatus globifer* sp. inc./*Hyalopomatus claparedii* sp. inc. (1071 ind. ha^−1^; 1.92% of all fauna) (Fig. 4a). All other fauna accounted for 4.83% of the total faunal density.

Co-occurrence network analysis of the faunal assemblage at CM showed no specific co-occurrences with the fish Gadidae fam. indet. and Liparidae fam. indet., Hexactinellida sponges, nor the gastropod Buccinidae fam. indet. (Fig. S2a). The analysis further indicated that Tetractinellida sponges co-occurred with a lower than expected frequency with the bivalve *Hyalopecten frigidus* (*p*_*lt*_ = 0.034) and with a higher than expected frequency with the anemone Hormathiidae (*p*_*gt*_ = 0.000) and polychaetes (*A. globifer*/*H. claparedii*, *p*_*gt*_ = 0.000) (Table S4).

A total of 852 specimens (1.53% of all fauna) were found associated with sponges, i.e., either as attached sessile epifauna or as mobile epifauna crawling over these, at CM. Among these, only five specimens were found associated with Hexactinellida sponges, with the remainder found on Tetractinellida specimens. Taxa most frequently associated with Tetractinellida gen. indet. were the shrimp *Bythocaris* sp. indet. (212 ind. ha^−1^; 25.1% of fauna associated with Tetractinellida gen. indet.), the anemone Hormathiidae gen. indet. (185 ind. ha^−1^; 21.8% of fauna associated with Tetractinellida gen. indet.), and the starfish *Tylaster willei* sp. inc. (180 ind. ha^−1^; 21.2% of fauna associated with Tetractinellida gen. indet.) (Fig. 4b–c,e–f).

#### Karasik Seamount

The faunal assemblage at KS was dominated by sponges of the order Tetractinellida (66.4% of all fauna; 33,422 ind. ha^−1^ sponges with < 8 cm diameter, 39,860 ind. ha^−1^ sponges with > 8 cm diameter; Fig. 4a,d) and the shrimp *Bythocaris* sp. indet. (22.9% of all fauna; 25,284 ind. ha^−1^; Fig. 4a,d, Table S4). The remaining faunal assemblage was predominantly composed by the mysid *Neobirsteiniamysis inermis* sp. inc. (4360 ind. ha^−1^), and the polychaete Macellicephalinae gen. indet. (2054 ind. ha^−1^) and *A. globifer* sp. inc./*H. claparedii* sp. inc. (1391 ind. ha^−1^). All other fauna accounted for 2.86% of the total faunal density.

**Figure 5:**
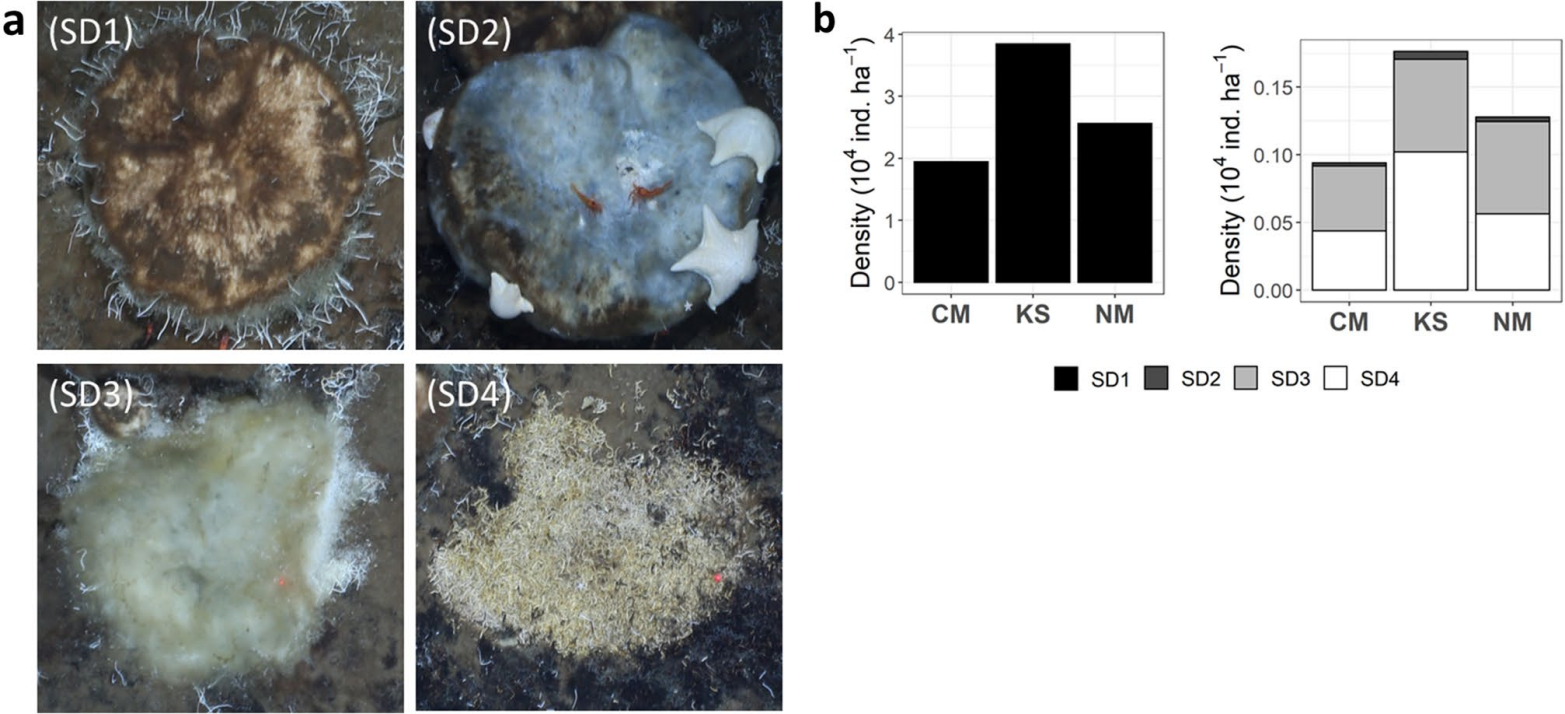
(**a**) Images of different stages of sponge decay observed across the three seamounts. (SD1) Healthy big Tetractinellida gen. indet. sponge, (SD2) Tetractinellida sponge partly covered with a white/blue microbial mat, (SD3) whitish-grey/bright yellow, collapsed sponge, (SD4) polychaetes covering the sponge remains. (**b**) Density of big sponges in different stages of decay/bleaching (in ca. 1000 specimens) across the three Arctic seamounts (*CM* Central Mount, *KS* Karasik Seamount, *NM* Northern Mount). For plotting purposes, densities of SD1 are shown in the left part of panel (**b**) and densities of SD2–SD4 are presented in the right part of panel (**b**).

The results of the co-occurrence network analysis of the assemblage at KS indicated that the brittle star *Ophiostriatus striatus* sp. inc., the fish *Lycodes* sp. indet., and Hexactinellida sponges had no specific co-occurrence with any other taxon in the faunal community (Fig. 5b). Tetractinellida sponges were observed to co-occur with gastropods Buccinidae fam. indet. at a lower frequency than expected by chance (*p*_*lt*_ = 0.014), and with polychaetes (Serpulidae and Siboglinidae, *p*_*gt*_ = 0.039; *A. globifer*/*H. claparedii*, *p*_*gt*_ = 0.000;) at a frequency higher than expected (Table S4).

A total of 6,244 specimens (5.66% of all fauna) were found associated with sponges, *i.e.*, either attached, crawling over or feeding on these, at KS. Among these, only 39 specimens were found associated to Hexactinellida sponges and the rest were on Tetractinellida sponges. Taxa most frequently associated with Tetractinellida gen. indet. were the shrimp *Bythocaris* sp. indet. (2975 ind. ha^−1^; 47.9% of fauna associated with Tetractinellida gen. indet.), the mysid *Neobirsteiniamysis inermis* sp. inc. (1129 ind. ha^−1^; 18.2% of fauna associated with Tetractinellida gen. indet.), the soft coral Nephtheidae gen. indet. (623 ind. ha^−1^; 10.0% of fauna associated with Tetractinellida gen. indet.), and juveniles of the sponge *Schaudinnia rosea* sp. inc. (527 ind. ha^−1^; 8.49% of fauna associated with Tetractinellida gen. indet.) (Fig. 4b–c,e–f).

#### Northern Mount

The faunal assemblage at NM was dominated by the sponge of the order Tetractinellida gen. indet. (73.9% of all fauna; 28,825 ind. ha^−1^ sponges with < 8 cm diameter, 20,054 ind. ha^−1^ sponges with > 8 cm diameter; Fig. 4a,d; Table S3) and the bryzoan Cyclostomatida fam. indet. (7469 ind. ha^−1^; 11.3% of all fauna). The remaining faunal assemblage was predominantly composed by the brittle star *Ophiostriatus striatus* sp. inc. (2380 ind. ha^−1^), the shrimp *Bythocaris* sp. indet. (2272 ind. ha^−1^), the starfish *Tylaster willei* sp. inc. (1435 ind. ha^−1^), and the polychaete Macellicephalinae gen. indet. (973 ind. ha^−1^). All other fauna accounted for 4.01% of the total faunal density.

Co-occurrence network analysis of the faunal assemblage at NM predicted no specific interactions between Hexactinellida sponges and any other megafaunal taxon at the seamount (Fig. S2b). Tetractinellida sponges co-occurred with a higher than expected frequency with anemones (Edwardsiidae gen. indet., *p*_*gt*_ = 0.000; Nephtheidae gen. indet., *p*_*gt*_ = 0.000), with polychaetes (Serpulidae and Siboglinidae, *p*_*gt*_ = 0.000; *A. globifer*/*H. claparedii*, *p*_*gt*_ = 0.000; Macellicephalinae gen. indet., *p*_*gt*_ = 0.000), and with the bryozoan Cyclostomatida fam. indet. (*p*_*gt*_ = 0.000) (Table S4). In comparison, Tetractinellida sponges at NM were observed to co-occur with a lower than expected frequency with fish (Liparidae fam. indet., *p*_*lt*_ = 0.004; *Lycodes* sp. indet., *p*_*lt*_ = 0.013; *Rhodichthys regina* inc., *p*_*lt*_ = 0.000) (Table S4).

A total of 1484 specimens (2.25% of all fauna) were found associated with sponges, i.e., either attached, crawling over or feeding on these, at NM. Among these, only 26 specimens were found associated to Hexactinellida sponges with the rest on Tetractinellida gen. indet. sponges. The taxa most frequently associated with Tetractinellida gen. indet., were the bryozoan Cyclostomatida fam. indet. (445 ind. ha^−1^, 30.5% of fauna associated with Tetractinellida gen. indet.), the starfish *Tylaster willei* sp. inc. (303 ind. ha^−1^, 20.8%), other small Tetractinellida gen. indet. specimens (167 ind. ha^−1^, 11.4%), and anemone Hormathiidae gen. indet. (161 ind. ha^−1^, 11.1%) (Fig. 4b–c,e–f).

#### Density of decaying sponges and their bulk and compound-specific isotope composition

At all three seamounts Tetractinellida gen. indet. sponges were observed in four different stages of decay (Fig. 5a). These decay stages ranged from healthy sponges (95.2–95.5% of all big Tetractinellida sponges; Fig. 5a SD1, b), to sponges that turned white/blue indicating the coverage with microbial mats (0.09–0.22% of all big Tetractinellida sponges; Fig. 5a SD2, b), to a whitish/bright yellow, collapsed sponge (1.79–2.54% of all big Tetractinellida sponges; Fig. 5a SD3, b), to dense mats of polychaetes covering the sponge remains (1.62–3.79% of all big Tetractinellida sponges; Fig. 5a SD4, b). Most sponges of decay stages SD1, SD2, and SD3 were observed at habitat type H4 at KS, while dense mats of polychaetes on top of sponge remains (SD4) were mostly observed at H4 at CM (Table S5).

To infer possible trophic interaction, in this study we used bulk isotopes (δ^13^C and δ^15^N) and phospholipid fatty acids (PLFAs) as organism-specific markers for the identification of food sources. At the Karasik Seamount, big Tetractinellida gen. indet. sponges had stable isotope values of (mean ± standard deviation) − 18.2 ± 0.22‰ δ^13^C and 8.16 ± 0.51‰ δ^15^N (*Geodia hentscheli*; n = 4), − 18.4 ± 0.15‰ δ^13^C and 8.43 ± 0.78‰ δ^15^N (*Geodia parva*; n = 7), and − 18.3 ± 0.36‰ δ^13^C and 8.42 ± 0.28‰ δ^15^N (*Geodia rhaphidiophora*; n = 3)^50^. Decaying sponges of stage SD2 had a stable isotopic composition of − 20.2 ± 0.45‰ δ^13^C and 4.82 ± 0.23‰ δ^15^N (n = 3) and the microbial mat that covered the sponges in decay stage SD2 had a stable isotopic composition of − 17.9 ± 0.08‰ δ^13^C and 10.8 ± 0.28‰ δ^15^N (n = 2). These microbial mats contained PLFAs that were to 74% bacteria-specific and 19% were sponge-specific PLFAs (Table S6).

Across the three seamounts, several taxa were observed in association with Tetractinellida sponges in different stages of decay, such as the asteroid *Tylaster willei* sp. inc., and the shrimps *Bythocaris* sp. indet. and *Neobirsteiniamysis inermis* sp. inc. Additionally, polychaetes of the family Macellicephalinae gen. indet. crawled over or fed upon decaying Tetractinellida sponges at the Karasik Seamount and byrozoans Cyclostomatida fam. indet. were associated with decaying Tetractinellida sponges at the Northern Mount. At the Karasik Seamount, these asteroids had a stable isotopic composition of − 13.4 ± 6.05‰ δ^13^C and 10.84 ± 1.53‰ δ^15^N (n = 2)^50^ and the total PLFA pool of a starfish collected from the top of a Tetractinellida sponge consisted to 48% of bacteria-specific PLFAs, to 33% of algae-specific PLFAs and to 6% of sponge-specific PLFAs (Table S6). Shrimps had stable isotope values of − 21.7 ± 1.71‰ δ^13^C and 12.4 ± 0.89‰ δ^15^N (n = 2)^50^. Unfortunately, due to lack of bulk material, no PLFAs were extracted from shrimps associated with the sponges, so no information about their PLFAs composition is available.

## Discussion

The seafloor at the three seamounts of the Langseth Ridge consisted mainly of bare rock, sand, and gravel along with a mix of biogenic structures composed of reef-forming sponge grounds, spicule mats, and polychaete tubes. Our results showed that the megafaunal densities and assemblage composition, but not taxon richness, differed at regional (between seamounts) and local (within seamount, between habitats) scales across the studied area. Demosponges of the order Tetractinellida numerically dominated the assemblages across the three seamounts, possibly owing to their unique capacity to source carbon directly from the refractory matter on the seabed^50^, which likely makes these mostly (if not fully) independent from the water column food-supply in such a low primary productivity area^71,72^. Shrimps (Central Mount, Karasik Seamount) and bryozoans (Northern Mount) were the other most abundant taxa encountered, yet by far not as abundant as by Tetractinellida sponges present in a much smaller area. Here, we discuss the potential processes causing the observed variations in megabenthic composition at different scales. We further elaborate on the functional role Tetractinellida sponges play in the high Arctic seamount ecosystem and describe a new possible pathway of the sponge loop as a potential additional mechanisms for recycling organic matter in this food-deprived ecosystem.

Variations in assemblage composition observed in regional assessments were likely related to inherent differences in megabenthos density across the three seamounts. This difference in densities could be correlated with the height of the seamounts: depth, or more precisely, the strong covariation of key factors (i.e., food supply and temperature) with increasing depth^73^ has been widely highlighted as major proxy for deep-sea benthic abundance and biomass^74,75^. As such, and in line with our results, many studies have shown how depth-related variations in population densities can yield markedly distinct benthic communities in seamounts^18,22,76^. Water temperature and current strength may be other drivers of the variations in faunal abundance observed. For instance, water temperatures measured at the Karasik Seamount (0.66 °C) and the Northern Mount (0.68 °C) were higher than at the Central Mount (0.23 °C)^77^. In contrast, current velocity measured during the cruise were generally weak (< 0.1 cm s^−1^) with a predominantly westwards component and no evidence of associated upwelling currents^78^. It is hence more plausible that food supply and temperature decreases with depth have a stronger influence on the observed variations in megabenthic abundance than the overlying current dynamics. It is noted that bottom currents and hydrographic processes can typically exhibit periodic or seasonal increases, leading to enhanced food supply rates (e.g.,^79^), particularly in interaction with the complex topography of seamounts^38^. However, we rule out the possibility that high densities of bryzoans at the Northern Mount was related to increased seasonal currents due to the sluggishness of the current over the year, and the year-round ice cover.

Variations in assemblage composition at local scale appears to be clearly driven by the existence of different habitats. Habitat type H4 (bare rock) and H5 (mixed substrate) covered between 87 and 89% of the seamount areas studied, whereupon H4 supported a relatively denser community compared to H5. This difference in megabenthic densities was partly related to variations in morphotype composition between H4 and H5 at the Northern Mount and the Central Mount. For instance, in both seamounts, only very few brittle stars were observed across bare rocks (H4), whereas they were very abundant across the mixed substrate seafloor areas (H5). The brittle star *Ophiostriatus striatus* is an opportunistic deposit feeder that was observed grazing upon fresh and detrital ice algae in the Nansen Basin close to the Gakkel Ridge during the minimum sea ice extent in 2012^80,81^. The mixed substrate (max thickness of spicule mat: 15 cm^50^) may trap settled particles^50^ which could subsequently serve as a food source for brittle stars and other deposit-feeding fauna. This would explain the very low densities of this brittle star across bare rocks where potentially increased hydrodynamics together with the lack of tridimensional structure provided by the spicule mat might prevent detritus accumulation. However, it remains unclear why it is almost absent from the Karasik Seamount as Zhulay and colleagues observed uncommon swimming behavior in the species^82^, which might facilitate the connectivity between the Central Mount and the Karasik Seamount. This suggests that a combination of regional and local environmental differences likely causes the variability between the megabenthos assemblages at the Northern and Central Mount and that at the Karasik Seamount.

Besides the habitat types H4 and H5, also H1 (Tetractinellida sponge grounds) and H3 (sediment with gravel) were observed at all seamounts, whereas H2 (mats of polychaete tubes) was found only at the Northern Mount and the Central Mount. However, owing to the primarily exploratory nature of the research cruise to investigate the geological, geochemical, and biological processes of the active hydrothermal vent at the Gakkel Ridge^83^ and seamounts at the Langseth Ridge in 2016^50,51,83^, the surrounding topography and community structure were not well known before the expedition. Therefore, no previous information was available to design a series of seabed image surveys that could grant a balanced sampling effort between habitat types (i.e. fully unknown prior to the expedition). Our bootstrap-based assessment allowed the reduction of this study limitation (*i.e.*, unbalanced sampling effort across different habitat types) by focusing on the variability associated to different ecological estimators rather than in the actual estimations (*e.g.*, mean values), which can be a robust way for instance, to infer ecological patterns in opportunistic deep-sea datasets (e.g.,^84,85^), yet was only conceived here as a preliminary approach. In this regard, the comparably smaller image sample size for H1, H2, and H3 did not allow for reliable statistical comparison of these ones with the more dominant H4 and H5. Thus, based on our preliminary work, future studies aimed at acquiring a better understanding of the community ecology and composition in this area shall now be able to appropriately design benthic image surveys, i.e., yielding even sampling effort across each of the, now characterized, Langseth Ridge habitat types.

The main habitat types of the deep Arctic Ocean are ridges, seeps, hydrothermal vents, and deep basins filled with soft sediment^81,86–91^. The megabenthic community observed at the Langseth Ridge differs substantially from the community at seeps and hydrothermal vents whose fauna is characterized by chemosynthetic taxa, such as siboglinid polychaetes^87–89,92^ and gastropods^91^, and from soft-sediment communities. These contain mostly the phyla Echinodermata, Cnidaria, Porifera, and Arthropoda^86,93,94^, and show a bathymetric distribution with a lower shelf/upper slope community (characteristic taxa: brittle star *Ophiocten sericeum*, bivalve *Yoldiella solidula*), a lower slope community (characteristic taxa: bivalve *Bathyarca frielei*, polychaete *Galathowenia fragilis*), and an abyssal community (characteristic taxa: polychaete *Anobothrus laubieri*, sea cucumber *Kolga hyalina*)^95^. Instead, the sponge grounds on the Central Mount, the Karasik Seamount, and the Northern Mount resemble partly the sponge ground community at the Schulz Bank seamount. The megafaunal community at this seamount comprised 20 taxa and was dominated by the Tetractinellida sponges *Geodia parva* and *Stelletta rhaphidiophora*^67^. Further abundant sessile taxa were ascidians, anthozoans (*Gersemia rubiformis*), other Demospongiae (*Lissodendoryx* (*Lissodendoryx*) *complicate*, *Hexadella dedritifera*), and Hexactinellida sponges^67^. Mobile taxa like echinoderms and fish were also observed, but they occurred at lower densities^67^.

Tetractinellida sponges at the Langseth Ridge host diverse taxa, such as juvenile sponges, anthozoans, byrozoans, or the polychaetes *A. globifer* sp. inc./*H. claparedii* sp. inc. The latter colonized the edge of Tetractinellida sponges as they may benefit from the water fluxes generated by the pumping activity of the sponges, as well as from the particle detritus expelled by them as a source of food for the epi-endobiota^96^. In comparison, only few specimens were found associated to hexactinellid sponges: several sponge specimens, bryozoans, and anthozoans. The difference in the number of associated specimens between the Hexactinellida and Tetractinellida sponges can be related to their different morphology (papillate/globular and massive, respectively) and the spicule “fur” produced by Tetractinellida that facilitate the epifauna settlement^61^. Additionally, Tetractinellida sponges at the Northern Mount, Karasik Seamount, or Central Mount had a large variety of mobile fauna associated with them, such as starfish, shrimps, and mysids. These starfishes either predate on the Tetractinellida sponges or graze upon sponge detritus, as sponge-specific PLFAs were detected in the analyzed starfish. Such predatory or detrital transfer of sponge-derived particulate organic matter (POM) to echinoderms has been measured in ex-situ pulse-chase incubation experiments by Bart et al.^68^. Using their experimental design with *Geodia barretti* and brittle stars, the authors were not able to differentiate between the so-called ‘deep-sea detrital sponge loop’ and the ‘deep-sea predatory sponge loop’^68^. In the “detrital sponge loop”, *G. barretti* released POM which is subsequently taken up by brittle stars^69^. On the other hand, in the ‘predatory sponge loop’, spongivores directly predate upon *G. barretti*.

**Figure 6:**
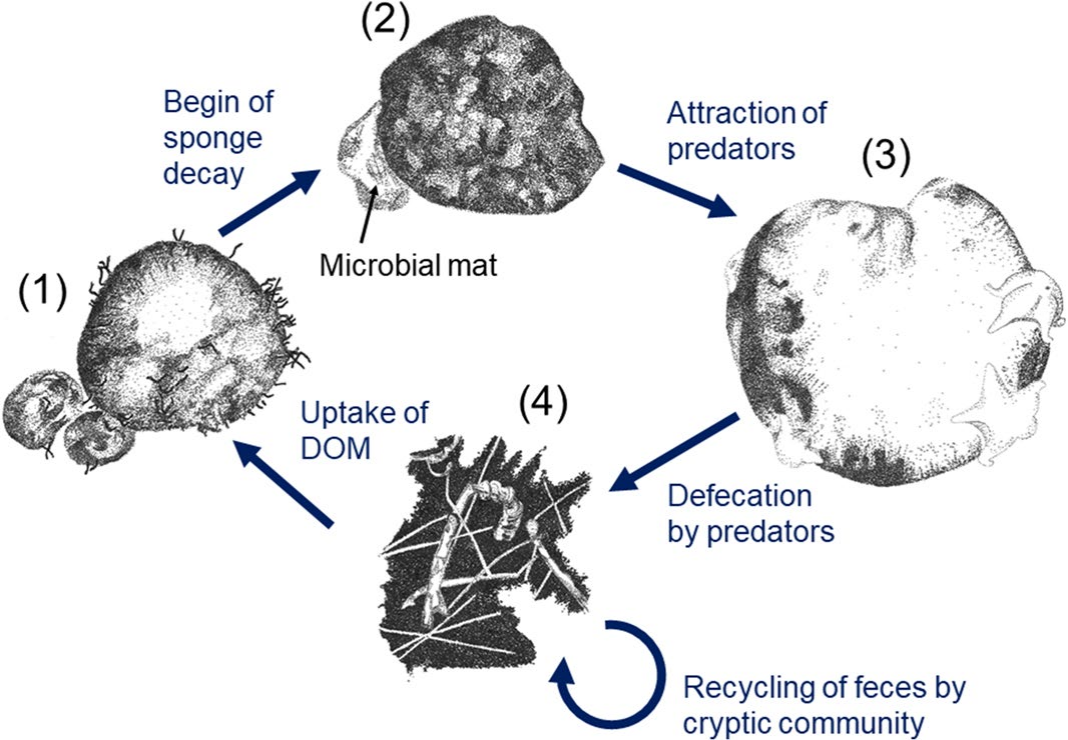
Conceptual model of the sponge loop likely present at the seamounts of the Langseth Ridge. The individual components of the sponge loop are the following: (1) Putatively healthy Tetractinellida sponges, (2) microbial mat covering a sponge, (3) asteroids predating upon the microbial mat covering a Tetractinellida sponge/ directly upon the sponge, (4) mat of polychaete tubes intermixed with sponge spicules hosting a cryptic microbial and faunal community. Illustrations by Tanja Stratmann.

In this study, we observed an additional pathway of the predatory deep-sea sponge loop that might function as follows (Fig. 6): an unknown chemical, physical, or biological cue triggers the decay of putatively healthy Tetractinellida sponges (δ^13^C value: − 18.2 to − 18.4‰)^50^. At the Tisler cold-water coral reef in Norway, for instance, a mass mortality event of *G. barretti* was partially related to temperature heat shocks^97,98^. During this event, the decaying sponges turned blue and black^97^, whereas (potentially bacterially-induced) diseased *G. barretti* from Korsfjord in Norway had a brown/black discoloration and disintegrated, fouling sponge tissue^98^. At the Langseth Ridge, decaying Tetractinellida sponges are covered by microbial mats and attract predators/ spongivores, such as starfishes. The starfish predates on the sponge as observed at the Schulz Bank seamount^99^ and/or grazes upon the microbial mat (δ^13^C value: − 17.9‰) covering the decaying sponge (δ^13^C value: − 20.2‰) as indicated by sponge-specific fatty acids and the higher δ^13^C value of − 13.4‰^50^. We hypothesize that feces produced by the starfish are subsequently recycled by the cryptic community living hidden in the sponge spicule-polychaete tube mats, such as amphipods, tanaidaceans, gastropods, and polychaetes^83^. The sponge spicule-polychaete tube mats may provide similar ecological and biogeochemical functions like ‘dead’ cold-water coral framework at Haas Mount in the North Atlantic^100^. There, dead framework increases resource retention and recycling like a “filtration-recycling factory”^100^. Maier et al.^100^ estimated that dead coral framework and living corals at Haas Mount had nearly equal contributions to the total oxygen consumption at the reef. At the Langseth Ridge, the cryptic community in the sponge spicule-polychaete tube mats likely releases dissolved organic matter (DOM) which can be taken up by the Tetractinellida sponges. Indeed, Bart et al.^68,101,102^ and Maier et al.^103^ measured dissolved organic carbon (DOC) uptake by *Geodia* sp. in ex-situ incubation experiments. This uptake of decaying sponge-derived DOM by healthy sponges would also support the relatively young age of Tetractinellida sponges that Morganti et al.^50^ reported in their study and related to the potential assimilation of relatively young DIC. We therefore propose such ‘deep-sea sponge loop’ as additional mechanism to re-cycle organic carbon matter in this ecosystem, facilitating the presence of such dense and abundant sponge community in the Langseth Ridge.

In conclusion, this study presents a detailed description of megabenthos assemblages at the northernmost seamounts explored so far. Interestingly, taxa richness did not differ between seamounts and habitats. While the megabenthos community composition showed substantial differences at regional and local scale, likely driven by intrinsic seamount characteristics (water temperature and depth) and distinct habitats, respectively. The Northern Mount had the highest density of bryozoans, which were almost absent in other seamounts and a more pronounced difference in megabenthic composition between the bare rock and mixed substrate habitats. So far, there is no evidence of particular processes, such as the increase of bottom currents or the different hydrographic conditions at the Northern Mount for explaining such distinctive features when compared to the other two seamounts. Further video and/or image transects at the individual seamounts are required to assess the megabenthos communities inhabiting the three other classified habitats.

Using bulk and compound-specific stable isotope analysis of phospholipid-derived fatty acids (PLFA) from Tetractinellida sponges, microbial mats, and starfish, this study showed the uptake of sponge-specific PLFA by starfishes that either originate from predation on the sponge (‘deep-sea predatory sponge loop’) or the uptake of sponge-derived detritus (‘deep-sea detrital sponge loop’). Starfish could also graze upon the microbial mat covering the decaying sponges. In either case, it is hypothesized that the feces of starfish are recycled by the cryptic community living in the sponge-spicule-polychaete tube mat and converted to DOM. This DOM may be subsequently taken up again by ‘healthy’ Tetractinellida sponges.

Like cold-water corals, sponges play an important role in habitat forming as ecosystem engineers and their spicules intermixed with the polychaete tubes create a perfect matrix for a “filtration-recycling factory”.

## Materials and methods

### Study area

**Figure 7:**
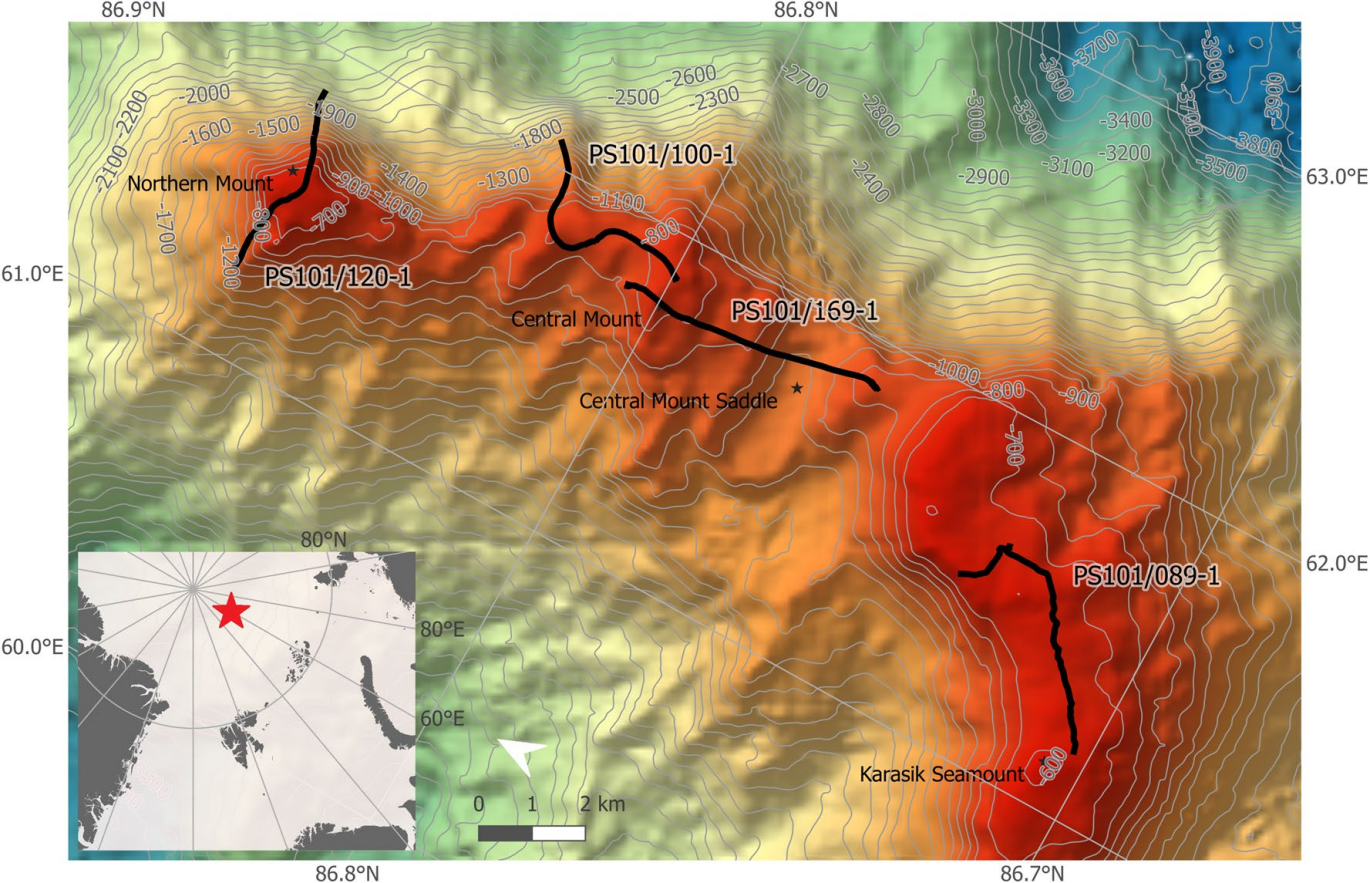
Map of the Northern Mount, Central Mount, and Karasik Seamount along the Langseth Ridge with all Ocean Floor Observation and Bathymetry System (OFOBS) deployments. The station numbers correspond to the OFOBS transect numbers in Table S7. The map was created using *ArcGIS* software, version 10.5 (https://www.arcgis.com).

The Langseth Ridge is a permanently ice-covered underwater mountain ridge in the central Arctic Ocean that stretches approximately 125 km from 87° N, 62° E to 85° 55′ N, 57.45′ E^83,104^ (Fig. 7). It is comprised of three summits, the Central Mount (CM), the Karasik Seamount (KS), and the Northern Mount (NM). The CM has its summit at 86° 47.83′ N, 61° 54.52′ E where its maximum elevation reaches to 722 m below the sea surface^83^. This seamount has a gradually increasing slope from 3300 m to its point of maximum elevation^83^. Its slope on the western side is steeper than the one on the eastern side where the slope drops to 4500 m water depth^83^. The tallest mountain on the Langseth Ridge is the KS which summit is located at 86° 43.0′ N, 61° 17.6′ E and reaches to 2500 m above the seafloor (i.e., 585 m below the sea surface^83^)^104^. The NM is located at 86° 51.86′ N, 61° 34′ E and has a maximum elevation of 631 m below the sea surface^83^. This seamount has a steep slope from its peak towards the Gakkel Ridge rift valley in the north at 4000 m depth^83^.

Bottom water at the three seamounts had a temperature range between − 0.02 °C (CM) and 0.22 °C (NM) and a salinity of 34.9 PSU^83^. Oxygen concentration in the water was only measured at the NM and amounted to 322 µmol L^−1^^105^. Nitrite was not detected in bottom water and ammonium was only found in KS’s bottom water (0.02 µmol L^−1^)^105^. Phosphate concentrations ranged in bottom water from 0.67 to 0.68 µmol L^−1^, nitrate concentration was between 12.0 and 12.7 µmol L^−1^, and silicate ranged from 5.53 to 5.86 µmol L^−1^^105^.

### Seabed image collection and processing

#### Image collection

The high-resolution digital photo camera (CANON EOS 5D Mark III, modified by iSiTEC for underwater applications) of the towed Ocean Floor Observation and Bathymetry System (OFOBS)^106^ was used to take still images of the seafloor of the three different seamounts. OFOBS was deployed four times (= four transects; Fig. 7 and Table S7) during the RV *Polarstern* cruise PS101 in the central Arctic Ocean (chief scientist: Prof. Dr. Antje Boetius)^83^. During each deployment, OFOBS was towed 1.5–2.5 m above the seafloor at a speed of < 1 knot and photographs were taken every 20 s to avoid overlap between images. The area of each image was calculated using three laser points on the seafloor that were organized in an equilateral triangle (distance between points: 0.5 m) as reference for scaling (area per image: mean ± SE: 8.43 ± 0.17 m^2^). A total of 3162 photographs were collected, from which 2099 (17,691 m^2^ of seabed) were used (Table S7), as only bright images collected within the aimed altitude range were selected for analysis. All photographs were loaded into the open-source software “Program for Annotation of Photographs and Rapid Analysis (of Zillions and Zillions) of Images” PAPARA(ZZ)I^107^.

#### Habitat classification

To classify macro- and microhabitat^108^ types at the different seamounts, it was recorded for each image whether $\ge$ 75% of the seafloor was covered by dense sponge grounds of *Geodia* sp. indet./*Stelletta* sp. indet. and sponge spicule mats (habitat type H1; includes parts of habitat category d in^50^), by mats of Serpulidae indet. and Siboglinidae indet. tubes covered with sulfide precipitates (habitat type H2; corresponds partly to habitat category b in^50^), by sediment with gravel (habitat type H3), or by bare rock (habitat type H4). When the seafloor was covered to $\le$ 75% by one of the four habitat types and therefore consisted of an assemblage of sponge ground and spicule mats, mats of polychaete tubes, sediment with gravel, and/ or bare rock, it was classified as mixed substrate (habitat type H5; habitat types a and c in^50^). The five habitat types described in this study are shown in Fig. 1.

#### Biological analysis

Megabenthic fauna (> 1 cm size; Fig. S1) visible on the photographs were annotated and identified to the lowest taxonomic hierarchy possible (morphotype [mtp]: typically Genus or Family level) based on previous image collection, published by^81^. Tetractinellida sponges were identified by spicule analysis in^50^ by Prof. Dr. Hans Thore Rapp. The taxonomic nomenclature of the morphotypes presented follows^109^.The life-habit of specimens was recorded whenever these were found attached (sessile fauna) or crawling (mobile fauna) on other specimens (i.e., generally sponges). As it is not possible to distinguish between *Geodia* sp. indet. and *Stelletta* sp. indet. sponges on seabed images, these specimens were identified as Tetractinellida gen. indet. (Fig. S1H). The latter were annotated based on their diameter size as “Tetractinellida gen. indet. ‘big’” (diameter > 8 cm) and “Tetractinellida gen. indet. ‘small’” (diameter < 8 cm). Polychaetes of the family Serpulidae indet. and Siboglinidae indet. (Fig. S1M) were not annotated individually, but as patches. The polychaetes *A. globifer* sp. inc/*H. claparedii* sp. inc (Fig. S1N) that were observed associated with Tetractinellida gen. indet. specimens were annotated as present/absent, i.e., they were annotated once per image when they were present. The uncertainty of the image-based identifications was indicated following the recommendations by^109^ for standardization of the open taxonomic nomenclature.

#### Quantitative data analysis

Patterns in diversity and distribution of faunal assemblages were quantitatively assessed at two scales: (i) regionally (scale: 10s km), across the three different seamounts (CM, KS, and NM), and (ii) locally (scale: 100s m), between the two habitats with the largest seafloor coverage (H4 and H5; encompassing 94% of all specimens and 88% of all the seabed area surveyed) across the three seamounts. In each case, megabenthos specimen data were pooled for each study area or target stratum, e.g., per seamount or habitat type, and then resampled using a modified form of bootstrapping^110^.

Resampling methods provide robust estimates of variability and confidence intervals of sample parameters^111,112^, and are particularly well suited to analyze seabed image data obtained from survey designs that lack true sample replication (see e.g.,^84,113^) like in this case with four OFOS transects across three seamounts due to the exploratory nature of the research cruise (Fig. 7 and Table S7). To implement the bootstrap, image data were randomly resampled with replacement until a minimum of 1000 megabenthos specimens were encompassed, and that process was repeated 10,000 times for each target stratum. This process yielded bootstrap-like samples (bootstrap generated sub-samples) with fixed specimen count size, ranging in total seabed cover from 72 to 490 m^2^, to minimize the potential effect of variable faunal densities in the estimation of ecological parameters.

A range of ecological parameters were calculated from each set of bootstrap-like samples to compare the assemblages from different target strata. Patterns in abundance were assessed by estimation of numerical density (ind. ha^−1^), whereas diversity was assessed by estimation of taxa richness (*S*, in ca. 1000 specimens) and Simpson’s index (*D*, in ca. 1000 specimens)^114^.

Variations in assemblage composition were assessed by non-metric multidimensional scaling (nMDS) ordination of bootstrap-like samples, based on the Bray–Curtis dissimilarity (or beta-diversity, β_BC_) measure^115^ calculated using square-root-transformed faunal density. Mean values of each parameter in each target stratum were calculated, along with corresponding C.I. 95% based on the simple percentile method^110^. All analyses were performed using a custom *R*^116^ script using multiple functions of the *vegan* package^117^.

Variations in ecological parameters between study areas were reported by comparing CI 95% (*i.e.*, the upper limit of a given estimate must be lower than the lower limits of the estimate that is compared to). Such cases are significant at *p* < 0.05, but the true (undetermined) *p*-value will, necessarily, be considerably lower.

Co-existence of taxa inhabiting the same seamount were investigated by a probabilistic model of co-occurrence^118^. For this purpose, records of taxa densities for each annotated seafloor image per seamount were converted into presence-absence records in order to perform the probabilistic taxa co-occurrence analysis for all images of a single seamount combined using the *cooccur* package^119^ in *R*. The resulting co-occurrence table reports the probability *p* of two taxa co-occurring at the same seamount with a higher frequency *p*_*gt*_ or lower frequency *p*_*lt*_ than observed^118^. When *p*_*gt*_ < 0.05, two taxa co-occur at a higher rate than expected by chance, and when *p*_*lt*_ < 0.05, two taxa co-occur at a lower rate than expected by chance^119^. For the CM dataset, 145 taxa pairs were investigated and 45 pairs (24% of all combinations) were excluded from the analysis because their co-occurrence was expected to be < 1. For the KS, 135 taxa pairs were analyzed and 55 pairs (29% of all combinations) were excluded, and for the NM, 147 taxa pairs were studied and 43 pairs (23% of all combinations) were discarded.

### Bulk and compound specific isotope analysis of microbial mats and sponge-associated fauna

Samples were obtained during RV *Polarstern* cruise PS101 from September to October 2016. Decaying sponges and bacteria mat were collected using push cores and the starfish observed on top of the sponge was collected using “Nereid Under Ice” remotely operated vehicle (NUI ROV). Samples were immediately stored on retrieval to the surface at − 20 °C for isotope analyses. Bulk stable isotope composition (δ^13^C, δ^15^N) of freeze-dried, pulverized star fish, microbial mat observed on top of a decaying sponge (Fig. 5a, SD2), and decaying sponge was measured on an elemental analyzer (EM) coupled with a Isotope Ratio Mass Spectrometer (IRMS) as described in^50^. PLFAs were extracted from freeze-dried, pulverized star fish and microbial mat following a modified Bligh and Dyer extraction^120^ as described in detail in the protocol by de Kluijver^121,122^.

## Supplementary Information

Supplementary Information.

## Data Availability

All “Ocean Floor Observation and Bathymetry System” (OFOBS) images collected during the RV *Polarstern* PS101 cruise are available at https://doi.pangaea.de/10.1594/PANGAEA.871550. All data generated and analyzed during this study are included in its supplementary information files.

## References

[1] Pitcher TJ (2007). Seamounts: Ecology, Fisheries & Conservation.

[2] Harris PT, Macmillan-Lawler M, Rupp J, Baker EK (2014). Geomorphology of the oceans. Mar. Geol..

[3] Wessel P, Sandwell DT, Kim S-S (2010). The global seamount census. Oceanography.

[4] Etnoyer PJ (2010). BOX 12|How large is the seamount biome?. Oceanography.

[5] De Forges BR, Koslow JA, Pooro GCB (2000). Diversity and endemism of the benthic seamount fauna in the southwest Pacific. Nature.

[6] Rowden AA, Dower JF, Schlacher TA, Consalvey M, Clark MR (2010). Paradigms in seamount ecology: Fact, fiction and future. Mar. Ecol..

[7] Pinheiro HT (2015). Fish biodiversity of the Vitória-Trindade seamount chain, southwestern Atlantic: An updated database. PLoS ONE.

[8] Morato T, Hoyle SD, Allain V, Nicol SJ (2010). Seamounts are hotspots of pelagic biodiversity in the open ocean. PNAS.

[9] Rowden AA (2010). A test of the seamount oasis hypothesis: Seamounts support higher epibenthic megafaunal biomass than adjacent slopes. Mar. Ecol..

[10] Busch K (2020). On giant shoulders: How a seamount affects the microbial community composition of seawater and sponges. Biogeosciences.

[11] Zhao Y (2020). Virioplankton distribution in the tropical western Pacific Ocean in the vicinity of a seamount. Microbiol Open.

[12] Arístegui J (2009). Plankton metabolic balance at two North Atlantic seamounts. Deep-Sea Res. II.

[13] Dower JF, Mackast DL (1996). “Seamount effects” in the zooplankton community near Cobb Seamount. Deep-Sea Res. I.

[14] O’Hara TD, Rowden AA, Bax NJ (2011). A Southern Hemisphere bathyal fauna is distributed in latitudinal bands. Curr. Biol..

[15] Williams A, Althaus F, Clark MR, Gowlett-Holmes K (2011). Composition and distribution of deep-sea benthic invertebrate megafauna on the Lord Howe Rise and Norfolk Ridge, southwest Pacific Ocean. Deep-Sea Res. II.

[16] Bridges AEH, Barnes DKA, Bell JB, Ross RE, Howell KL (2021). Benthic assemblage composition of South Atlantic seamounts. Front. Mar. Sci..

[17] Lapointe AE, Watling L, France SC, Auster PJ (2020). Megabenthic assemblages in the lower bathyal (700–3000 m) on the New England and corner rise seamounts Northwest Atlantic. Deep-Sea Res. I.

[18] Clark MR, Bowden DA (2015). Seamount biodiversity: High variability both within and between seamounts in the Ross Sea region of Antarctica. Hydrobiologia.

[19] McClain CR, Lundsten L, Barry J, DeVogelaere A (2010). Assemblage structure, but not diversity or density, change with depth on a northeast Pacific seamount. Mar. Ecol..

[20] Long DJ, Baco AR (2014). Rapid change with depth in megabenthic structure-forming communities of the Makapu’u deep-sea coral bed. Deep-Sea Res. II.

[21] Thresher R (2014). Strong septh-related zonation of megabenthos on a rocky continental margin (∼ 700–4000 m) off southern Tasmania Australia. PLoS ONE.

[22] O’Hara TD, Consalvey M, Lavrado HP, Stocks KI (2010). Environmental predictors and turnover of biota along a seamount chain. Mar. Ecol..

[23] Boschen RE (2015). Megabenthic assemblage structure on three New Zealand seamounts: Implications for seafloor massive sulfide mining. Mar. Ecol. Prog. Ser..

[24] Caratori Tontini F (2012). Crustal magnetization of brothers volcano, New Zealand, measured by autonomous underwater vehicles: Geophysical expression of a submarine hydrothermal system. Econ. Geol..

[25] Rex MA, Etter RJ, Clain AJ, Hill MS (1999). Bathymetric patterns of body size in deep-sea gastropods. Evolution (N Y).

[26] O’Hara TD (2007). Seamounts: Centres of endemism or species richness for ophiuroids?. Glob. Ecol. Biogeogr..

[27] Clark MR (2010). The ecology of seamounts: Structure, function, and human impacts. Ann. Rev. Mar. Sci..

[28] Cowen RK, Sponaugle S (2009). Larval dispersal and marine population connectivity. Ann. Rev. Mar. Sci..

[29] Levin LA, Thomas CL (1989). The influence of hydrodynamic regime on infaunal assemblages inhabiting carbonate sediments on central Pacific seamounts. Deep Sea Res. A.

[30] Puerta P (2022). Variability of deep-sea megabenthic assemblages along the western pathway of the Mediterranean outflow water. Deep-Sea Res. I.

[31] Tapia-Guerra JM (2021). First description of deep benthic habitats and communities of oceanic islands and seamounts of the Nazca Desventuradas Marine Park Chile. Sci. Rep..

[32] Morgan NB, Goode S, Roark EB, Baco AR (2019). Fine scale assemblage structure of benthic invertebrate megafauna on the North Pacific seamount Mokumanamana. Front. Mar. Sci..

[33] Perez JAA, Kitazato H, Sumida PYG, Sant’Ana R, Mastella AM (2018). Benthopelagic megafauna assemblages of the Rio Grande Rise (SW Atlantic). Deep-Sea Res. I.

[34] Poore GCB (2015). Invertebrate diversity of the unexplored marine western margin of Australia: Taxonomy and implications for global biodiversity. Mar. Biodivers..

[35] Henry LA, Moreno Navas J, Roberts JM (2013). Multi-scale interactions between local hydrography, seabed topography, and community assembly on cold-water coral reefs. Biogeosciences.

[36] Meyer KS (2016). Rocky islands in a sea of mud: Biotic and abiotic factors structuring deep-sea dropstone communities. Mar. Ecol. Prog. Ser..

[37] Stratmann T, Soetaert K, Kersken D, van Oevelen D (2021). Polymetallic nodules are essential for food-web integrity of a prospective deep-seabed mining area in Pacific abyssal plains. Sci. Rep..

[38] Genin A, Dayton PK, Lonsdale PF, Spiess FN (1986). Corals on seamount peaks provide evidence of current acceleration over deep-sea topography. Nature.

[39] Roberts JM, Wheeler AJ, Freiwald A (2006). Reefs of the deep: The biology and geology of cold-water coral ecosystems. Science.

[40] Kutti T, Bannister RJ, Fosså JH (2013). Community structure and ecological function of deep-water sponge grounds in the Traenadypet MPA-Northern Norwegian continental shelf. Cont. Shelf Res..

[41] Beazley L, Kenchington EL, Murillo FJ, Sacau MDM (2013). Deep-sea sponge grounds enhance diversity and abundance of epibenthic megafauna in the Northwest Atlantic. ICES J. Mar. Sci..

[42] Buhl-Mortensen L (2010). Biological structures as a source of habitat heterogeneity and biodiversity on the deep ocean margins. Mar. Ecol..

[43] Victorero L, Robert K, Robinson LF, Taylor ML, Huvenne VAI (2018). Species replacement dominates megabenthos beta diversity in a remote seamount setting. Sci. Rep..

[44] Yesson C, Clark MR, Taylor ML, Rogers AD (2011). The global distribution of seamounts based on 30 arc seconds bathymetry data. Deep-Sea Res. I.

[45] ICES. *Report of the ICES-NAFO Working Group on Deep-Water Ecology (WGDEC), 9–13 March 2009, ICES CM2009\ACOM:23*. *2009*.

[46] Cárdenas P, Rapp HT (2015). Demosponges from the Northern mid-Atlantic ridge shed more light on the diversity and biogeography of North Atlantic deep-sea sponges. J. Mar. Biol. Assoc. U.K..

[47] Cárdenas P (2013). Taxonomy, biogeography and DNA barcodes of Geodia species (Porifera, Demospongiae, Tetractinellida) in the Atlantic boreo-arctic region. Zool. J. Linn. Soc..

[48] Roberts EM (2018). Oceanographic setting and short-timescale environmental variability at an Arctic seamount sponge ground. Deep-Sea Res. I.

[49] Roberts E (2021). Water masses constrain the distribution of deep-sea sponges in the North Atlantic Ocean and Nordic seas. Mar. Ecol. Prog. Ser..

[50] Morganti TM (2022). Giant sponge grounds of central Arctic seamounts are associated with extinct seep life. Nat. Commun..

[51] Morganti TM (2021). In situ observation of sponge trails suggests common sponge locomotion in the deep central Arctic. Curr. Biol..

[52] Meyer HK, Roberts EM, Rapp HT, Davies AJ (2019). Spatial patterns of arctic sponge ground fauna and demersal fish are detectable in autonomous underwater vehicle (AUV) imagery. Deep-Sea Res. I.

[53] McIntyre FD, Drewery J, Eerkes-Medrano D, Neat FC (2016). Distribution and diversity of deep-sea sponge grounds on the Rosemary bank seamount NE Atlantic. Mar. Biol..

[54] Buhl-Mortensen P, Buhl-Mortensen L (2014). Diverse and vulnerable deep-water biotopes in the Hardangerfjord. Mar. Biol. Res..

[55] de Clippele LH (2018). The effect of local hydrodynamics on the spatial extent and morphology of cold-water coral habitats at Tisler Reef Norway. Coral Reefs.

[56] Dunlop K, Harendza A, Plassen L, Keeley N (2020). Epifaunal habitat Associations on mixed and hard bottom substrates in coastal waters of Northern Norway. Front. Mar. Sci..

[57] Fiore CL, Cox Jutte P (2010). Characterization of macrofaunal assemblages associated with sponges and tunicates collected off the southeastern United States. Biology.

[58] Murillo FJ (2012). Deep-sea sponge grounds of the Flemish Cap, Flemish Pass and the Grand Banks of Newfoundland (Northwest Atlantic Ocean): Distribution and species composition. Mar. Biol. Res..

[59] Purser A (2013). Local variation in the distribution of benthic megafauna species associated with cold-water coral reefs on the Norwegian margin. Cont. Shelf Res..

[60] Klitgaard AB, Tendal OS (2004). Distribution and species composition of mass occurrences of large-sized sponges in the northeast Atlantic. Prog. Oceanogr..

[61] Klitgaard AB (1995). The fauna associated with outer shelf and upper slope sponges (porifera, demospongiae) at the faroe islands, northeastern Atlantic. Sarsia.

[62] Cárdenas P, Moore JA (2019). First records of Geodia demosponges from the New England seamounts, an opportunity to test the use of DNA mini-barcodes on museum specimens. Mar. Biodivers..

[63] Schejter L, Chiesa IL, Doti BL, Bremec C (2012). Mycale (Aegogropila) magellanica (Porifera: Demospongiae) in the southwestern Atlantic Ocean: Endobiotic fauna and new distributional information. Sci. Mar..

[64] Beaulieu SE (2001). Life on glass houses: Sponge stalk communities in the deep sea. Mar. Biol..

[65] Goren L, Idan T, Shefer S, Ilan M (2021). Macrofauna inhabiting massive demosponges from shallow and mesophotic habitats along the Israeli Mediterranean coast. Front. Mar. Sci..

[66] Kersken D (2014). The infauna of three widely distributed sponge species (Hexactinellida and Demospongiae) from the deep Ekström Shelf in the Weddell Sea Antarctica. Deep-Sea Res. II.

[67] Meyer HK, Roberts EM, Rapp HT, Davies AJ (2019). Spatial patterns of arctic sponge ground fauna and demersal fish are detectable in autonomous underwater vehicle (AUV) imagery. Deep Sea Res. 1 Oceanogr. Res. Pap..

[68] Bart MC, Hudspith M, Rapp HT, Verdonschot PFM, de Goeij JM (2021). A Deep-Sea Sponge Loop? Sponges transfer dissolved and particulate organic carbon and nitrogen to associated fauna. Front. Mar. Sci..

[69] de Goeij JM (2013). Surviving in a marine desert: The sponge loop retains resources within coral reefs. Science.

[70] Pawlik, J. R. & Mcmurray, S. E. The emerging ecological and biogeochemical importance of sponges on coral reefs. (2019) 10.1146/annurev-marine-010419 10.1146/annurev-marine-010419-010807 31226028

[71] Wassmann P, Slagstad D, Ellingsen I (2010). Primary production and climatic variability in the European sector of the Arctic Ocean prior to 2007: Preliminary results. Polar Biol..

[72] Arrigo KR, van Dijken G, Pabi S (2008). Impact of a shrinking Arctic ice cover on marine primary production. Geophys. Res. Lett..

[73] Dunne JP, Sarmiento JL, Gnanadesikan A (2007). A synthesis of global particle export from the surface ocean and cycling through the ocean interior and on the seafloor. Glob. Biogeochem. Cycles.

[74] Wei C-L (2010). Global patterns and predictions of seafloor biomass using random forests. PLoS ONE.

[75] Stratmann T (2020). The BenBioDen database, a global database for meio-, macro- and megabenthic biomass and densities. Sci. Data.

[76] McClain CR, Lundsten L, Ream M, Barry J, DeVogelaere A (2009). Endemicity, biogeography, composition, and community structure on a Northeast Pacific seamount. PLoS ONE.

[77] Walter M, Köhler J, Myriel H, Steinmacher B, Wisotzki A (2017). PANGAEA.

[78] van Appen W-J, Latarius K, Kanzow T (2017). PANGAEA.

[79] Ruhl HA, Smith KL (2004). Shifts in deep-sea community structure linked to climate and food supply. Science.

[80] Boetius A (2013). Export of algal biomass from the melting Arctic sea ice. Science.

[81] Rybakova E, Kremenetskaia A, Vedenin A, Boetius A, Gebruk A (2019). Deep-sea megabenthos communities of the Eurasian Central Arctic are influenced by ice-cover and sea-ice algal falls. PLoS ONE.

[82] Zhulay I, Bluhm BA, Renaud PE, Degen R, Iken K (2021). Functional pattern of benthic epifauna in the Chukchi borderland Arctic deep sea. Front. Mar. Sci..

[83] Boetius A, Purser A (2017). The expedition PS101 of the research vessel Polarstern to the Arctic Ocean in 2016. Berichte zur Polar-und Meeresforschung = Rep Polar Mar Res.

[84] Simon-Lledó E (2020). Multi-scale variations in invertebrate and fish megafauna in the mid-eastern Clarion Clipperton Zone. Prog. Oceanogr..

[85] Simon-Lledó E (2019). Preliminary observations of the abyssal megafauna of Kiribati. Front. Mar. Sci..

[86] Zhulay I, Iken K, Renaud PE, Bluhm BA (2019). Epifaunal communities across marine landscapes of the deep Chukchi Borderland (Pacific Arctic). Deep Sea Res. 1 Oceanogr. Res. Pap..

[87] Åström EKL, Sen A, Carroll ML, Carroll JL (2020). Cold seeps in a warming Arctic: Insights for benthic ecology. Front. Mar. Sci..

[88] Pedersen RB (2010). Discovery of a black smoker vent field and vent fauna at the Arctic Mid-Ocean Ridge. Nat. Commun..

[89] Åström EKL (2018). Methane cold seeps as biological oases in the high-Arctic deep sea. Limnol. Oceanogr..

[90] Rybakova Goroslavskaya E, Galkin S, Bergmann M, Soltwedel T, Gebruk A (2013). Density and distribution of megafauna at the Håkon Mosby mud volcano (the Barents Sea) based on image analysis. Biogeosciences.

[91] Sweetman AK, Levin LA, Rapp HT, Schander C (2013). Faunal trophic structure at hydrothermal vents on the southern mohn’s ridge, arctic ocean. Mar. Ecol. Prog. Ser..

[92] Decker C, Olu K (2010). Does macrofaunal nutrition vary among habitats at the Hakon Mosby mud volcano?. Cah. Biol. Mar..

[93] Macdonald IR, Bluhm BA, Iken K, Gagaev S, Strong S (2010). Benthic macrofauna and megafauna assemblages in the Arctic deep-sea Canada Basin. Deep-Sea Res. II.

[94] Taylor J, Krumpen T, Soltwedel T, Gutt J, Bergmann M (2017). Dynamic benthic megafaunal communities: Assessing temporal variations in structure, composition and diversity at the Arctic deep-sea observatory HAUSGARTEN between 2004 and 2015. Deep Sea Res. 1 Oceanogr. Res. Pap..

[95] Vedenin AA (2022). Uniform bathymetric zonation of marine benthos on a Pan-Arctic scale. Prog. Oceanogr..

[96] Bart MC (2021). A deep-sea sponge loop? Sponges transfer dissolved and particulate organic carbon and nitrogen to associated fauna. Front. Mar. Sci..

[97] Guihen D, White M, Lundälv T (2014). Temperature shocks and ecological implications at a cold-water coral reef. ANZIAM J..

[98] Strand R (2017). The response of a boreal deep-sea sponge holobiont to acute thermal stress. Sci. Rep..

[99] Hanz U (2022). The important role of sponges in carbon and nitrogen cycling in a deep-sea biological hotspot. Funct. Ecol..

[100] Maier SR (2021). Reef communities associated with ‘dead’ cold-water coral framework drive resource retention and recycling in the deep sea. Deep-Sea Res. I.

[101] Bart MC (2020). Dissolved organic carbon (DOC) is essential to balance the metabolic demands of four dominant North-Atlantic deep-sea sponges. Limnol. Oceanogr..

[102] Bart MC (2020). Differential processing of dissolved and particulate organic matter by deep-sea sponges and their microbial symbionts. Sci. Rep..

[103] Maier SR (2020). Recycling pathways in cold-water coral reefs: Use of dissolved organic matter and bacteria by key suspension feeding taxa. Sci. Rep..

[104] International Hydrographic Bureau. 16th meeting of the GEBCO sub-committee on undersea feature names (SCUFN). Preprint at (2003).

[105] Torres-Valdés S, Morische A, Wischnewski L (2019). PANGAEA.

[106] Purser A (2019). Ocean floor observation and bathymetry system (OFOBS): A new towed camera/sonar system for deep-sea habitat surveys. IEEE J. Ocean. Eng..

[107] Marcon Y, Purser A (2017). PAPARA(ZZ)I : An open-source software interface for annotating photographs of the deep-sea. SoftwareX.

[108] Greene HG, Bizzarro JJ, O’Connell VM, Brylinsky CK (2007). Construction of digital potential marine benthic habitat maps using a coded classification scheme and its application. Spec. Pap.: Geol. Assoc. Canada.

[109] Horton T (2021). Recommendations for the standardisation of open taxonomic nomenclature for image-based identifications. Front. Mar. Sci..

[110] Davison AC, Hinkley DV (1997). Bootstrap Methods and Their Application.

[111] Rodgers JL (1999). The bootstrap, the jackknife, and the randomization test: A sampling taxonomy. Multivar. Behav. Res..

[112] Crowley PH (1992). Resampling methods for computation-intensive data analysis in ecology and evolution. Annu. Rev. Ecol. Syst..

[113] Simon-Lledó E (2019). Ecology of a polymetallic nodule occurrence gradient: Implications for deep-sea mining. Limnol. Oceanogr..

[114] Jost L (2006). Entropy and diversity. Oikos.

[115] Clarke KR (1993). Non-parametric multivariate analyses of changes in community structure. Aust. J. Ecol..

[116] R-Core Team. R: A language and environment for statistical computing. Preprint at https://www.r-project.org/ (2017).

[117] Oksanen, J. *et al.* vegan: Community ecology package. Preprint at (2017).

[118] Veech JA (2013). A probabilistic model for analysing species co-occurrence. Glob. Ecol. Biogeogr..

[119] Griffith DM, Veech JA, Marsh CJ (2016). Cooccur: Probabilistic species co-occurrence analysis in R. J. Stat. Softw..

[120] Bligh EG, Dyer WJ (1959). A rapid method of total lipid extraction and purification. Can. J. Biochem. Physiol..

[121] de Kluijver A (2021). Fatty acid analysis sponges. protocols.io.

[122] de Kluijver A (2021). Bacterial precursors and unsaturated long-chain fatty acids are biomarkers of North-Atlantic deep-sea demosponges. PLoS ONE.

