# Supplementary material for: Habitat types and megabenthos composition from three sponge-dominated high-Arctic seamounts
Source: Sci Rep. 2022 Nov 29;12:20610. doi: 10.1038/s41598-022-25240-z (PMC9708660; doi:10.1038/s41598-022-25240-z)
Supplement: Supplementary file 1 — Supplementary Information. [file 41598_2022_25240_MOESM1_ESM.docx]

**Supplementary Information for “****Habitat types and megabenthos composition from three sponge-dominated high-Arctic seamounts”**

**Tanja Stratmann^1,2,3^*, Erik Simon-Lledó^4^, Teresa Maria Morganti^2,5^, Anna de Kluijver**^1^**, Andrey Vedenin^6^, Autun Purser^7^**

^1^ Department of Earth Sciences, Utrecht University, Vening Meineszgebouw A, Princetonlaan 8, 3584 CB Utrecht, The Netherlands.

^2^ HGF MPG Joint Research Group for Deep-Sea Ecology and Technology, Max Planck Institute for Marine Microbiology, Celsiusstraße 1, 28359 Bremen, Germany.

^3^ Department of Ocean Systems, NIOZ – Royal Netherlands Institute for Sea Research, Landsdiep 4, 1797 SZ ‘t Horntje (Texel), The Netherlands.

^4^ Ocean BioGeosciences, National Oceanography Centre, European Way, Southampton SO14 3ZH, UK.

^5^ Marine Chemistry Department, Leibniz Institute for Baltic Sea Research Warnemünde, Seestraße 15, 18119 Rostock, Germany.

^6^ Marine Biology Section, Senckenberg am Meer, Südstrand 40, 26382 Wilhelmshaven, Germany.

^7^ Alfred Wegener Institute, Helmholtz Centre for Polar and Marine Research, Am Handelshafen 12, 27570 Bremerhaven, Germany.

**Table S1.** Seabed coverage (m^2^ and %) and total number of specimens observed at each different seamount and habitat type. Habitat types are presented in Fig. 1.

| **Seamount** | **Habitat type** | **Images** | **Seabed coverage** | **Specimens** |
| --- | --- | --- | --- | --- |
| Central Mount | H1 | 44 | 514 (7%) | 730 |
|  | H2 | not observed | not observed | not observed |
|  | H3 | 62 | 436 (6%) | 772 |
|  | H4 | 525 | 4,061 (56%) | 25,566 |
|  | H5 | 332 | 2,245 (31%) | 6,482 |
| Karasik Seamount | H1 | 77 | 512 (10%) | 2,523 |
|  | H2 | 3 | 11 (0.2%) | 55 |
|  | H3 | 8 | 29 (1%) | 105 |
|  | H4 | 588 | 3,933 (73%) | 44,405 |
|  | H5 | 148 | 874 (16%) | 6,668 |
| Northern Mount | H1 | 24 | 217 (4%) | 2,359 |
|  | H2 | 3 | 63 (1%) | 125 |
|  | H3 | 15 | 365 (7%) | 226 |
|  | H4 | 186 | 1,553 (31%) | 16,776 |
|  | H5 | 84 | 2,879 (57%) | 2,848 |

**Table S2.** Average density (95% confidence intervals; ind. ha^-1^) of invertebrate megafauna and fish specimens observed at habitats H4 (bare rock) and H5 (mixed substrate) at the different seamounts.

| **Phylum, Class** | **Taxon** | **Central Mount** | | **Karasik Seamount** | | **Northern Mount** | |
| --- | --- | --- | --- | --- | --- | --- | --- |
|  |  | **H4** | **H5** | **H4** | **H5** | **H4** | **H5** |
| Annelida, Polychaeta | *Apomatus globifer* sp. inc./ *Hyalopomatus claparedii* sp. inc. | 1,081  (818 – 1337) | 1,162  (940 – 1,398) | 1,362  (1,027 –1,747) | 1,624  (1,301 – 1,946) | 954  (510 – 1,594) | 131  (69 – 214) |
|  | Macellicephalinae gen. indet. | 304  (57 – 660) | 357  (152 – 603) | 1,858  (752 – 3,568) | 2,793  (1,677 – 4,074) | 1,376  (369 – 3,102) | 247  (109 – 446) |
|  | Serpulidae gen. indet. and Siboglinidae gen. indet. | 350  (56 – 808) | 438  (166 – 765) | 561  (0 – 1,388) | 1,424  (605 – 2,468) | 327  (0 – 889) | 74  (12 – 167) |
| Arthropoda, Malacostraca | *Bythocaris* sp. indet. | 4,337  (2,779 – 6,144) | 2,538  (1,711 – 3,471) | 27,511  (16,823 – 43,486) | 20,554  (15,429 – 26,395) | 4,105  (2,436 – 5,671) | 715  (395 – 1,167) |
|  | *Neobirsteiniamysis inermis* sp. inc. | 517  (126 – 1,059) | 198  (30 – 450) | 4,659  (1,930 – 8,434) | 4,228  (2,229 – 6,838) | 159  (0 – 502) | 35  (0 – 92) |
| Bryozoa, Stenolaemata | Cyclostomatida fam. indet. | 21  (0 – 132) | 24  (0 – 103) | 70  (0 – 315) | 370  (0 – 904) | 9,141  (1,608 – 2,3811) | 1,910  (408 – 4,380) |
| Chordata, Actinopteri | Gadidae fam. indet. | 3  (0 – 62) | not observed | 12  (0 – 122) | not observed | 17  (0 – 114) | not observed |
|  | Liparidae fam. indet. | 31  (0 – 171) | 32  (0 – 107) | 7  (0 – 108) | 10  (0 – 86) | 10  (0 – 103) | 41  (0 – 94) |
|  | *Lycodes* sp. indet. | 71  (0 – 238) | 59  (0 – 157) | not observed | not observed | not observed | 18  (0 – 59) |
|  | *Rhodichthys regina* inc. | 69  (0 – 225) | 37  (0 – 132) | 30  (0 – 192) | 17  (0 – 92) | 5  (0 – 90) | 26  (0 – 80) |
| Cnidaria, Anthozoa | Edwardsiidae gen. indet. | 47  (0 – 192) | 60  (0 – 168) | 190  (0 – 605) | 210  (0 – 557) | 97  (0 – 355) | 18  (0 – 52) |
|  | Hormathiidae gen. indet. | 457  (126 – 885) | 158  (31 – 321) | 779  (211 – 1,565) | 567  (213 – 1,021) | 445  (73 – 1,322) | 257  (125 – 431) |
|  | Nephtheidae gen. indet. | 86  (0 – 383) | 31  (0 – 122) | 785  (0 – 2,582) | 794  (158 – 1,782) | 326  (0 – 1,371) | 71  (0 – 186) |
| Echinodermata, Asteroidea | *Tylaster willei* sp. inc. | 793  (173 – 3,090) | 218  (62 – 418) | 484  (0 – 1,178) | 509  (137 – 1,003) | 2,610  (911 – 5,152) | 291  (92 – 617) |
| Echinodermata, Ophiuroidea | *Ophiostriatus striatus* sp. inc. | 534  (63 – 1,484) | 1,904  (790 – 3,713) | not observed | 30  (0 – 160) | 956  (113 – 4,782) | 3,888  (2,101 – 5,992) |
| Mollusca, Bivalvia | *Hyalopecten frigidus* sp. inc. | 111  (0 – 434) | 349  (67 – 898) | 3  (0 – 97) | 22  (0 – 142) | 9  (0 – 110) | 214  (0 – 829) |
| Mollusca, Gastropoda | Buccinidae fam. indet. | 42  (0 – 192) | 106  (0 – 244) | 123  (0 – 576) | 379  (0–1,369) | 12  (0 – 169) | 61  (0 – 176) |
| Porifera, Demospongiae | Tetractinellida gen. indet. ‘small’ | 804  (342 – 1,370) | 355  (132 – 647) | 853  (252 – 1,652) | 463  (124 – 924) | 929  (328 – 1,764) | 67  (10 – 154) |
|  | Tetractinellida gen. indet. ‘big’ | 61,544  (48,512 – 77,619) | 26,490  (20,732 – 33,831) | 81,488  (64,584 – 102,912) | 47,934  (37,275 – 60,246) | 91,514  (65,501 – 123,525) | 5,689  (2,698 – 10,384) |
| Porifera, Hexactinellida | *Hyalonema* sp. indet. | 41  (0 – 300) | not observed | 64  (0 – 590) | 10  (0 – 85) | 35  (0 – 213) | 48  (0 – 121) |
|  | *Schaudinnia rosea* sp. inc. | 36  (0 – 167) | 56  (0 – 154) | 47  (0 – 263) | 130  (0 – 385) | 8  (0 – 103) | 162  (21 – 402) |
|  | *Schaudinnia rosea* sp. inc. juv. | 82  (0 – 274) | 82  (0 – 202) | 558  (95 – 1,271) | 1,014  (377 – 1,895) | 83  (0 – 524) | 160  (14 – 495) |

**Table S3.** Average density (95% confidence intervals; ind. ha^-1^) of invertebrate megafauna and fish specimens observed at the different seamounts.

| **Phylum, Class** | **Taxon** | **Central Mount** | **Karasik Seamount** | **Northern Mount** |
| --- | --- | --- | --- | --- |
| Annelida, Polychaeta | *Apomatus globifer* sp. inc./ *Hyalopomatus claparedii* sp. inc. | 1,071  (820 – 1,331) | 1,391  (1,049 – 1,769) | 536  (233 – 1094) |
|  | Macellicephalinae gen. indet. | 325  (91 – 641) | 2,054  (929 – 3,628) | 973  (262 – 2,379) |
|  | Serpulidae gen. indet. and Siboglinidae gen. indet. | 355  (64 – 760) | 700  (103 – 1,579) | 245  (0 – 767) |
| Arthropoda, Malacostraca | *Bythocaris* sp. indet. | 3,502  (2,256 – 4,984) | 25,284  (16,151 – 38,945) | 2,272  (1,004 – 4,277) |
|  | *Neobirsteiniamysis inermis* sp. inc. | 380  (92 – 795) | 4,360  (1,926 – 7,798) | 86  (0 – 315) |
| Bryozoa, Stenolaemata | Cyclostomatida fam. indet. | 20  (0 – 111) | 132  (0 – 486) | 7,469  (1,028 – 21,417) |
| Chordata, Actinopteri | Gadidae fam. indet. | 2  (0 – 46) | 11  (0 – 110) | 8  (0 – 71) |
|  | Liparidae fam. indet. | 31  (0 – 149) | 8  (0 – 102) | 26  (0 – 124) |
|  | *Lycodes* sp. indet. | 66  (0 – 206) | not observed | 9  (0 – 87) |
|  | *Rhodichthys regina* inc. | 58  (0 – 190) | 26  (0 – 160) | 22  (0 – 134) |
| Cnidaria, Anthozoa | Edwardsiidae gen. indet. | 51  (0 – 179) | 188  (0 – 571) | 99  (0 – 473) |
|  | Hormathiidae gen. indet. | 346  (91 – 695) | 711  (198 – 1,431) | 657  (73 – 4,107) |
|  | Nephtheidae gen. indet. | 62  (0 – 282) | 745  (81 – 2,347 | 589  (0 – 2,835) |
| Echinodermata, Asteroidea | *Tylaster willei* sp. inc. | 622  (137 – 2,364) | 492  (85 – 1,128) | 1,453  (422 – 3,406) |
| Echinodermata, Ophiuroidea | *Ophiostriatus striatus* sp. inc. | 1,133  (239 – 2,796) | 4  (0 – 95) | 2,380  (345 – 5,906) |
| Mollusca, Bivalvia | *Hyalopecten frigidus* sp. inc. | 180  (0 – 665) | 10  (0 – 107) | 76  (0 – 1,255) |
| Mollusca, Gastropoda | Buccinidae fam. indet. | 59  (0 – 203) | 163  (0 – 1,211) | 30  (0 – 233) |
| Porifera, Demospongiae | Tetractinellida gen. indet. | 47,334 (35,961 – 62,380) | 73,256 (56,615 – 93,726) | 48,753 (22,584 – 91,922) |
| Porifera, Hexactinellida | *Hyalonema* sp. indet. | 29  (0 – 232) | 51  (0 – 478) | 42  (0 – 186) |
|  | *Schaudinnia rosea* sp. inc. | 114  (0 – 289) | 661  (116 – 1,448) | 217  (0 – 1,060) |

**Table S4.** Co-occurrence tables of all significant interactions between taxa pairs whose co-occurrence was expected to be >1 at a specific seamount. p  0.001: ⁂, p  0.01: ⁑, p  0.05: *

Abbreviations: No image_taxon 1_ = number of images where taxon 1 was observed, No image_taxon 2_ = number of images where taxon 2 was observed, No image_taxon 1+2_ = number of images where both taxa were observed, P_co-occurrence_ = probability that both species were observed in an image, E_co-occurrence_ = expected number of images with both taxa, *p_gt_ =* probabilty of co-occurrence of both taxa at a higher frequency than observed, *p_lt_* = probabilty of co-occurrence of both taxa at lower frequency than observed.

| **Taxon 1** | **Taxon 2** | **No image_taxon 1_** | | **No image_taxon 2_** | | **No image_taxon 1+2_** | | **P_co-occurrence_** | | **E_co-occurrence_** | | ***p_lt_*** | | ***p_gt_*** |
| --- | --- | --- | --- | --- | --- | --- | --- | --- | --- | --- | --- | --- | --- | --- |
| **Central Mount** | | | | | | | | | | | | | | |
| *Tylaster willei* | *Bythocaris* sp. | 200 | | 664 | | 161 | | 0.150 | | 141.1 | | 1.000 | | **0.000⁂** |
| *Tylaster willei* | *Neobirsteiniamysis inermis* | 200 | | 153 | | 44 | | 0.035 | | 32.5 | | 0.994 | | **0.010⁑** |
| *Tylaster willei* | *Hyalopecten frigidus* | 200 | | 89 | | 10 | | 0.020 | | 18.9 | | **0.008⁑** | | 0.997 |
| *Tylaster willei* | Hormathiidae | 200 | | 167 | | 52 | | 0.038 | | 35.5 | | 1.000 | | **0.001⁂** |
| *Ophiostriatus striatus* | *Bythocaris* sp. | 291 | | 664 | | 187 | | 0.218 | | 205.3 | | **0.003⁑** | | 0.998 |
| *Ophiostriatus striatus* | Tetractinellida | 291 | | 892 | | 261 | | 0.293 | | 0.293 | | **0.000⁂** | | 1.000 |
| *Ophiostriatus striatus* | Hormathiidae | 291 | | 167 | | 33 | | 0.055 | | 51.6 | | **0.000⁂** | | 1.000 |
| *Ophiostriatus striatus* | *Apomatus globifer*/ *Hyalopomatus claparedii* | 291 | | 820 | | 242 | | 0.269 | | 253.6 | | **0.011⁑** | | 0.994 |
| *Ophiostriatus striatus* | Cyclostomatida | 291 | | 12 | | 7 | | 0.004 | | 3.7 | | 0.989 | | **0.044*** |
| *Ophiostriatus striatus* | Macellicephalinae | 291 | | 191 | | 71 | | 0.063 | | 59.1 | | 0.985 | | **0.023*** |
| *Bythocaris* sp. | *Neobirsteiniamysis inermis* | 664 | | 153 | | 144 | | 0.115 | | 108.0 | | 1.000 | | **0.000⁂** |
| *Bythocaris* sp. | *Schaudinnia rosea* | 664 | | 66 | | 59 | | 0.049 | | 46.6 | | 1.000 | | **0.000⁂** |
| *Bythocaris* sp. | Tetractinellida | 664 | | 892 | | 655 | | 0.669 | | 629.4 | | 1.000 | | **0.000⁂** |
| *Bythocaris* sp. | *Hyalopecten frigidus* | 664 | | 89 | | 50 | | 0.067 | | 62.8 | | **0.002⁑** | | 1.000 |
| *Bythocaris* sp. | Hormathiidae | 664 | | 167 | | 147 | | 0.125 | | 117.8 | | 1.000 | | **0.000⁂** |
| *Bythocaris* sp. | Edwardsiidae | 664 | | 41 | | 22 | | 0.031 | | 28.9 | | **0.014⁑** | | 0.994 |
| *Bythocaris* sp. | *Apomatus globifer*/ *Hyalopomatus claparedii* | 664 | | 820 | | 610 | | 0.615 | | 578.6 | | 1.000 | | **0.000⁂** |
| *Bythocaris* sp. | Nephtheidae | 664 | | 38 | | 36 | | 0.028 | | 26.8 | | 1.000 | | **0.000⁂** |
| *Neobirsteiniamysis inermis* | *Schaudinnia rosea* | 153 | | 66 | | 19 | | 0.011 | | 10.7 | | 0.998 | | **0.006⁑** |
| *Neobirsteiniamysis inermis* | Tetractinellida | 153 | | 892 | | 153 | | 0.154 | | 145.0 | | 1.000 | | **0.000⁂** |
| *Neobirsteiniamysis inermis* | *Hyalopecten frigidus* | 153 | | 89 | | 7 | | 0.015 | | 14.5 | | **0.013⁑** | | 0.995 |
| *Neobirsteiniamysis inermis* | Hormathiidae | 153 | | 167 | | 39 | | 0.029 | | 27.2 | | 0.997 | | **0.006⁑** |
| *Neobirsteiniamysis inermis* | *Lycodes* sp. | 153 | | 45 | | 3 | | 0.008 | | 7.3 | | **0.047*** | | 0.986 |
| *Schaudinnia rosea* | Tetractinellida | 66 | | 892 | | 66 | | 0.066 | | 62.6 | | 1.000 | | **0.026*** |
| *Schaudinnia rosea* | Hormathiidae | 66 | | 167 | | 19 | | 0.012 | | 11.7 | | 0.993 | | **0.015*** |
| Tetractinellida | *Hyalopecten frigidus* | 892 | | 89 | | 80 | | 0.090 | | 84.4 | | **0.034*** | | 0.987 |
| Tetractinellida | Hormathiidae | 892 | | 167 | | 167 | | 0.168 | | 158.3 | | 1.000 | | **0.000⁂** |
| Tetractinellida | *Apomatus globifer*/ *Hyalopomatus claparedii* | 892 | | 820 | | 818 | | 0.826 | | 777.3 | | 1.000 | | **0.000⁂** |
| *Hyalopecten frigidus* | Edwardsiidae | 89 | | 41 | | 10 | | 0.004 | | 3.9 | | 0.999 | | **0.003⁑** |
| *Hyalopecten frigidus* | *Lycodes* sp. | 89 | | 45 | | 9 | | 0.005 | | 4.3 | | 0.993 | | **0.020*** |
| *Hyalopecten frigidus* | Macellicephalinae | 89 | | 191 | | 8 | | 0.019 | | 18.1 | | **0.002⁑** | | 0.999 |
| Hormathiidae | *Apomatus globifer*/ *Hyalopomatus claparedii* | 167 | | 820 | | 157 | | 0.155 | | 145.5 | | 1.000 | | **0.001⁂** |
| Hormathiidae | *Rhodichthys regina* | 167 | | 35 | | 12 | | 0.007 | | 6.2 | | 1.000 | | **0.013⁑** |
| Hormathiidae | *Lycodes* sp. | 167 | | 45 | | 16 | | 0.008 | | 8.0 | | 0.999 | | **0.003⁑** |
| Serpulidae and Siboglinidae | *Apomatus globifer*/ *Hyalopomatus claparedii* | 188 | | 820 | | 151 | | 0.174 | | 163.8 | | **0.002⁑** | | 0.999 |
| *Apomatus globifer*/ *Hyalopomatus claparedii* | *Rhodichthys regina* | 820 | | 35 | | 35 | | 0.032 | | 30.5 | | 1.000 | | **0.007⁑** |
| *Apomatus globifer*/ *Hyalopomatus claparedii* | Nephtheidae | 820 | | 38 | | 29 | | 0.035 | | 33.1 | | **0.045*** | | 0.983 |
| Cyclostomatida | Macellicephalinae | 12 | | 191 | | 6 | | 0.003 | | 2.4 | | 0.996 | | **0.020*** |
| **Karasik Seamount** | | | | | | | | | | | | | | |
| *Tylaster willei* | *Neobirsteiniamysis inermis* | | 189 | | 586 | | 144 | | 0.164 | | 134.6 | | 0.967 | **0.050*** |
| *Bythocaris* sp. | *Neobirsteiniamysis inermis* | | 819 | | 586 | | 586 | | 0.709 | | 583.2 | | 1.000 | **0.007⁑** |
| *Neobirsteiniamysis inermis* | *Schaudinnia rosea* | | 586 | | 246 | | 188 | | 0.213 | | 175.2 | | 0.988 | **0.018*** |
| *Neobirsteiniamysis inermis* | Tetractinellida | | 586 | | 814 | | 586 | | 0.704 | | 579.6 | | 1.000 | **0.000⁂** |
| *Neobirsteiniamysis inermis* | Hormathiidae | | 586 | | 249 | | 192 | | 0.215 | | 177.3 | | 0.995 | **0.008⁑** |
| *Neobirsteiniamysis inermis* | Liparidae | | 586 | | 5 | | 1 | | 0.004 | | 3.6 | | **0.026*** | 0.998 |
| *Neobirsteiniamysis inermis* | Cyclostomatida | | 586 | | 63 | | 52 | | 0.055 | | 44.9 | | 0.989 | **0.024*** |
| *Neobirsteiniamysis inermis* | Nephtheidae | | 586 | | 185 | | 146 | | 0.160 | | 131.7 | | 0.997 | **0.005⁑** |
| *Schaudinnia rosea* | Buccinidae | | 246 | | 51 | | 23 | | 0.019 | | 15.2 | | 0.994 | **0.013*** |
| *Schaudinnia rosea* | Serpulidae and Siboglinidae | | 246 | | 248 | | 92 | | 0.090 | | 74.1 | | 0.999 | **0.002⁑** |
| *Schaudinnia rosea* | *Apomatus globifer*/ *Hyalopomatus claparedii* | | 246 | | 772 | | 238 | | 0.280 | | 230.8 | | 0.995 | **0.013*** |
| *Schaudinnia rosea* | Cyclostomatida | | 246 | | 63 | | 27 | | 0.023 | | 18.8 | | 0.992 | **0.016*** |
| Tetractinellida | Buccinidae | | 814 | | 51 | | 48 | | 0.061 | | 50.4 | | **0.014*** | 0.999 |
| Tetractinellida | Serpulidae and Siboglinidae | | 814 | | 248 | | 248 | | 0.298 | | 245.3 | | 1.000 | **0.039*** |
| Tetractinellida | *Apomatus globifer*/ *Hyalopomatus claparedii* | | 814 | | 772 | | 772 | | 0.928 | | 763.6 | | 1.000 | **0.000⁂** |
| *Hyalopecten frigidus* | Serpulidae and Siboglinidae | | 6 | | 248 | | 5 | | 0.002 | | 1.8 | | 0.999 | **0.011⁑** |
| Hormathiidae | Serpulidae and Siboglinidae | | 249 | | 248 | | 62 | | 0.091 | | 75.0 | | **0.018*** | 0.988 |
| Hormathiidae | *Rhodichthys regina* | | 249 | | 12 | | 10 | | 0.004 | | 3.6 | | 1.000 | **0.000⁂** |
| Hormathiidae | Gadidae | | 249 | | 5 | | 4 | | 0.002 | | 1.5 | | 0.998 | **0.031*** |
| Hormathiidae | Nephtheidae | | 249 | | 185 | | 67 | | 0.068 | | 56.0 | | 0.981 | **0.029*** |
| Edwardsiidae | Nephtheidae | | 87 | | 185 | | 27 | | 0.024 | | 19.6 | | 0.982 | **0.033*** |
| Serpulidae and Siboglinidae | Cyclostomatida | | 248 | | 63 | | 37 | | 0.023 | | 19.0 | | 1.000 | **0.000⁂** |
| Serpulidae and Siboglinidae | Macellicephalinae | | 248 | | 544 | | 180 | | 0.199 | | 163.9 | | 0.996 | **0.006⁑** |
| *Apomatus globifer*/ *Hyalopomatus claparedii* | *Rhodichthys regina* | | 772 | | 12 | | 2 | | 0.014 | | 11.3 | | **0.000⁂** | 1.000 |
| *Apomatus globifer*/ *Hyalopomatus claparedii* | Cyclostomatida | | 772 | | 63 | | 63 | | 0.072 | | 59.1 | | 1.000 | **0.015*** |
| Cyclostomatida | Macellicephalinae | | 63 | | 544 | | 48 | | 0.051 | | 41.6 | | 0.974 | **0.05*** |
| **Northern Mount** | | | | | | | | | | | | | | |
| *Tylaster willei* | *Ophiostriatus striatus* | | 204 | | 153 | | 77 | | 0.327 | | 101.0 | | **0.000⁂** | 1.000 |
| *Tylaster willei* | *Bythocaris* sp. | | 204 | | 225 | | 161 | | 0.481 | | 148.5 | | 1.000 | **0.000⁂** |
| *Tylaster willei* | *Neobirsteiniamysis inermis* | | 204 | | 29 | | 24 | | 0.062 | | 19.1 | | 1.000 | **0.032*** |
| *Tylaster willei* | *Schaudinnia rosea* | | 204 | | 51 | | 19 | | 0.109 | | 33.7 | | **0.000⁂** | 1.000 |
| *Tylaster willei* | *Hyalonema* sp. | | 204 | | 16 | | 4 | | 0.034 | | 10.6 | | **0.001⁂** | 1.000 |
| *Tylaster willei* | Tetractinellida | | 204 | | 235 | | 190 | | 0.502 | | 155.1 | | 1.000 | **0.000⁂** |
| *Tylaster willei* | *Hyalopecten frigidus* | | 204 | | 9 | | 2 | | 0.019 | | 5.9 | | **0.008⁑** | 0.999 |
| *Tylaster willei* | Buccinidae | | 204 | | 11 | | 3 | | 0.024 | | 7.3 | | **0.009⁑** | 0.999 |
| *Tylaster willei* | Serpulidae and Siboglinidae | | 204 | | 63 | | 54 | | 0.135 | | 41.6 | | 1.000 | **0.000⁂** |
| *Tylaster willei* | *Apomatus globifer*/ *Hyalopomatus claparedii* | | 204 | | 231 | | 188 | | 0.494 | | 152.5 | | 1.000 | **0.000⁂** |
| *Tylaster willei* | *Lycodes* sp. | | 204 | | 5 | | 1 | | 0.011 | | 3.3 | | **0.047*** | 0.996 |
| *Tylaster willei* | Liparidae | | 204 | | 16 | | 4 | | 0.034 | | 10.6 | | **0.001⁂** | 1.000 |
| *Tylaster willei* | Cyclostomatida | | 204 | | 184 | | 151 | | 0.393 | | 121.5 | | 1.000 | **0.000⁂** |
| *Tylaster willei* | Macellicephalinae | | 204 | | 184 | | 141 | | 0.393 | | 121.5 | | 1.000 | **0.000⁂** |
| *Tylaster willei* | Nephtheidae | | 204 | | 56 | | 55 | | 0.120 | | 37.0 | | 1.000 | **0.000⁂** |
| *Ophiostriatus striatus* | *Schaudinnia rosea* | | 153 | | 51 | | 36 | | 0.082 | | 25.3 | | 1.000 | **0.001⁂** |
| *Ophiostriatus striatus* | *Hyalonema* sp. | | 153 | | 16 | | 13 | | 0.026 | | 7.9 | | 1.000 | **0.008⁑** |
| *Ophiostriatus striatus* | Tetractinellida | | 153 | | 235 | | 91 | | 0.377 | | 116.4 | | **0.000⁂** | 1.000 |
| *Ophiostriatus striatus* | Buccinidae | | 153 | | 11 | | 9 | | 0.018 | | 5.4 | | 0.995 | **0.028*** |
| *Ophiostriatus striatus* | *Apomatus globifer*/ *Hyalopomatus claparedii* | | 153 | | 231 | | 87 | | 0.370 | | 114.4 | | **0.000⁂** | 1.000 |
| *Ophiostriatus striatus* | *Rhodichthys regina* | | 153 | | 10 | | 9 | | 0.016 | | 5.0 | | 0.999 | **0.009⁑** |
| *Ophiostriatus striatus* | Liparidae | | 153 | | 16 | | 15 | | 0.026 | | 7.9 | | 1.000 | **0.000⁂** |
| *Ophiostriatus striatus* | Cyclostomatida | | 153 | | 184 | | 65 | | 0.295 | | 91.1 | | **0.000⁂** | 1.000 |
| *Ophiostriatus striatus* | Macellicephalinae | | 153 | | 184 | | 82 | | 0.295 | | 91.1 | | **0.023*** | 0.987 |
| *Ophiostriatus striatus* | Nephtheidae | | 153 | | 56 | | 16 | | 0.090 | | 27.7 | | **0.000⁂** | 1.000 |
| *Bythocaris* sp. | *Neobirsteiniamysis inermis* | | 225 | | 29 | | 28 | | 0.068 | | 21.1 | | 1.000 | **0.001⁂** |
| *Bythocaris* sp. | *Schaudinnia rosea* | | 225 | | 51 | | 30 | | 0.120 | | 37.1 | | **0.013⁑** | 0.995 |
| *Bythocaris* sp. | Tetractinellida | | 225 | | 235 | | 186 | | 0.554 | | 171.1 | | 1.000 | **0.000⁂** |
| *Bythocaris* sp. | Buccinidae | | 225 | | 11 | | 5 | | 0.026 | | 8.0 | | **0.047*** | 0.989 |
| *Bythocaris* sp. | *Apomatus globifer*/ *Hyalopomatus claparedii* | | 225 | | 231 | | 187 | | 0.544 | | 168.2 | | 1.000 | **0.000⁂** |
| *Bythocaris* sp. | Cyclostomatida | | 225 | | 184 | | 143 | | 0.434 | | 134.0 | | 0.993 | **0.014⁑** |
| *Bythocaris* sp. | Macellicephalinae | | 225 | | 184 | | 142 | | 0.434 | | 134.0 | | 0.986 | **0.026*** |
| *Neobirsteiniamysis inermis* | Tetractinellida | | 29 | | 235 | | 28 | | 0.071 | | 22.1 | | 1.000 | **0.003⁑** |
| *Neobirsteiniamysis inermis* | *Apomatus globifer*/ *Hyalopomatus claparedii* | | 29 | | 231 | | 28 | | 0.070 | | 21.7 | | 1.000 | **0.002⁑** |
| *Neobirsteiniamysis inermis* | Cyclostomatida | | 29 | | 184 | | 25 | | 0.056 | | 17.3 | | 1.000 | **0.001⁂** |
| *Schaudinnia rosea* | *Hyalonema* sp. | | 51 | | 16 | | 8 | | 0.009 | | 2.6 | | 1.000 | **0.001⁂** |
| *Schaudinnia rosea* | Tetractinellida | | 51 | | 235 | | 19 | | 0.126 | | 38.8 | | **0.000⁂** | 1.000 |
| *Schaudinnia rosea* | *Hyalopecten frigidus* | | 51 | | 9 | | 4 | | 0.005 | | 1.5 | | 0.992 | **0.044*** |
| *Schaudinnia rosea* | *Apomatus globifer*/ *Hyalopomatus claparedii* | | 51 | | 231 | | 19 | | 0.123 | | 38.1 | | **0.000⁂** | 1.000 |
| *Schaudinnia rosea* | *Rhodichthys regina* | | 51 | | 10 | | 6 | | 0.005 | | 1.7 | | 1.000 | **0.002⁑** |
| *Schaudinnia rosea* | Liparidae | | 51 | | 16 | | 6 | | 0.009 | | 2.6 | | 0.992 | **0.032*** |
| *Schaudinnia rosea* | Cyclostomatida | | 51 | | 184 | | 8 | | 0.098 | | 30.4 | | **0.000⁂** | 1.000 |
| *Schaudinnia rosea* | Macellicephalinae | | 51 | | 184 | | 15 | | 0.098 | | 30.4 | | **0.000⁂** | 1.000 |
| *Schaudinnia rosea* | Nephtheidae | | 51 | | 56 | | 2 | | 0.030 | | 9.2 | | **0.001⁂** | 1.000 |
| *Hyalonema* sp. | Tetractinellida | | 16 | | 235 | | 5 | | 0.039 | | 12.2 | | **0.000⁂** | 1.000 |
| *Hyalonema* sp. | *Apomatus globifer*/ *Hyalopomatus claparedii* | | 16 | | 231 | | 6 | | 0.039 | | 12.0 | | **0.001⁂** | 1.000 |
| *Hyalonema* sp. | Cyclostomatida | | 16 | | 184 | | 0 | | 0.031 | | 9.5 | | **0.000⁂** | 1.000 |
| Tetractinellida | Edwardsiidae | | 235 | | 27 | | 27 | | 0.066 | | 20.5 | | 1.000 | **0.000⁂** |
| Tetractinellida | Serpulidae and Siboglinidae | | 235 | | 63 | | 58 | | 0.155 | | 47.9 | | 1.000 | **0.000⁂** |
| Tetractinellida | *Apomatus globifer*/ *Hyalopomatus claparedii* | | 235 | | 231 | | 230 | | 0.569 | | 175.7 | | 1.000 | **0.000⁂** |
| Tetractinellida | *Rhodichthys regina* | | 235 | | 10 | | 2 | | 0.025 | | 7.6 | | **0.000⁂** | 1.000 |
| Tetractinellida | *Lycodes* sp. | | 235 | | 5 | | 1 | | 0.012 | | 3.8 | | **0.013⁑** | 0.999 |
| Tetractinellida | Liparidae | | 235 | | 16 | | 7 | | 0.039 | | 12.2 | | **0.004⁑** | 0.999 |
| Tetractinellida | Cyclostomatida | | 235 | | 184 | | 173 | | 0.453 | | 139.9 | | 1.000 | **0.000⁂** |
| Tetractinellida | Macellicephalinae | | 235 | | 184 | | 154 | | 0.453 | | 139.9 | | 1.000 | **0.000⁂** |
| Tetractinellida | Nephtheidae | | 235 | | 56 | | 56 | | 0.138 | | 42.6 | | 1.000 | **0.000⁂** |
| *Hyalopecten frigidus* | Macellicephalinae | | 9 | | 184 | | 2 | | 0.017 | | 5.4 | | **0.025*** | 0.996 |
| Buccinidae | *Apomatus globifer*/ *Hyalopomatus claparedii* | | 11 | | 231 | | 5 | | 0.027 | | 8.2 | | **0.033*** | 0.993 |
| Hormathiidae | Cyclostomatida | | 97 | | 184 | | 65 | | 0.187 | | 57.8 | | 0.974 | **0.045*** |
| Hormathiidae | Nephtheidae | | 97 | | 56 | | 26 | | 0.057 | | 17.6 | | 0.997 | **0.007⁑** |
| Edwardsiidae | Serpulidae and Siboglinidae | | 27 | | 63 | | 10 | | 0.018 | | 5.5 | | 0.991 | **0.028*** |
| Edwardsiidae | *Apomatus globifer*/ *Hyalopomatus claparedii* | | 27 | | 231 | | 27 | | 0.065 | | 20.2 | | 1.000 | **0.000⁂** |
| Edwardsiidae | Cyclostomatida | | 27 | | 184 | | 22 | | 0.052 | | 16.1 | | 0.997 | **0.011⁑** |
| Edwardsiidae | Macellicephalinae | | 27 | | 184 | | 22 | | 0.052 | | 16.1 | | 0.997 | **0.011⁑** |
| Edwardsiidae | Nephtheidae | | 27 | | 56 | | 10 | | 0.016 | | 4.9 | | 0.997 | **0.012⁑** |
| Serpulidae and Siboglinidae | *Apomatus globifer*/ *Hyalopomatus claparedii* | | 63 | | 231 | | 58 | | 0.152 | | 47.1 | | 1.000 | **0.000⁂** |
| Serpulidae and Siboglinidae | Liparidae | | 63 | | 16 | | 0 | | 0.011 | | 3.3 | | **0.023*** | 1.000 |
| Serpulidae and Siboglinidae | Cyclostomatida | | 63 | | 184 | | 52 | | 0.121 | | 37.5 | | 1.000 | **0.000⁂** |
| Serpulidae and Siboglinidae | Nephtheidae | | 63 | | 56 | | 17 | | 0.037 | | 11.4 | | 0.985 | **0.035*** |
| *Apomatus globifer*/ *Hyalopomatus claparedii* | *Rhodichthys regina* | | 231 | | 10 | | 2 | | 0.024 | | 7.5 | | **0.000⁂** | 1.000 |
| *Apomatus globifer*/ *Hyalopomatus claparedii* | *Lycodes* sp. | | 231 | | 5 | | 1 | | 0.012 | | 3.7 | | **0.015*** | 0.999 |
| *Apomatus globifer*/ *Hyalopomatus claparedii* | Liparidae | | 231 | | 16 | | 7 | | 0.039 | | 12.0 | | **0.007⁑** | 0.996 |
| *Apomatus globifer*/ *Hyalopomatus claparedii* | Cyclostomatida | | 231 | | 184 | | 171 | | 0.445 | | 137.6 | | 1.000 | **0.000⁂** |
| *Apomatus globifer*/ *Hyalopomatus claparedii* | Macellicephalinae | | 231 | | 184 | | 153 | | 0.445 | | 137.6 | | 1.000 | **0.000⁂** |
| *Apomatus globifer*/ *Hyalopomatus claparedii* | Nephtheidae | | 231 | | 56 | | 55 | | 0.135 | | 41.9 | | 1.000 | **0.000⁂** |
| *Rhodichthys regina* | Cyclostomatida | | 10 | | 184 | | 1 | | 0.019 | | 6.0 | | **0.002⁑** | 1.000 |
| *Lycodes* sp. | Cyclostomatida | | 5 | | 184 | | 0 | | 0.010 | | 3.0 | | **0.010⁑** | 1.000 |
| Liparidae | Cyclostomatida | | 16 | | 184 | | 4 | | 0.031 | | 9.5 | | **0.004⁑** | 0.999 |
| Liparidae | Nephtheidae | | 16 | | 56 | | 0 | | 0.009 | | 2.9 | | **0.037*** | 1.000 |
| Cyclostomatida | Macellicephalinae | | 184 | | 184 | | 128 | | 0.355 | | 109.6 | | 1.000 | **0.000⁂** |
| Cyclostomatida | Nephtheidae | | 184 | | 56 | | 55 | | 0.108 | | 33.3 | | 1.000 | **0.000⁂** |
| Macellicephalinae | Nephtheidae | | 184 | | 56 | | 51 | | 0.108 | | 33.3 | | 1.000 | **0.000⁂** |

**Table S5.** Average density (95% confidence intervals; ind. ha^-1^) of big Tetractinellida sponges present in four stages of decay (SD1, SD2, SD3, SD4; Fig. 6) that were observed at habitat typess H1, H2, H3, H4, and H5 (Fig. 1) at the different seamounts.

| **Sponge decay stage** | **H1** | **H2** | **H3** | **H4** | **H5** |
| --- | --- | --- | --- | --- | --- |
| **Central Mount** | | | | | |
| SD1 | 3,992  (0 – 23,883) | not observed | 3,945  (0 – 17,624) | 27,762  (3,112 – 72,386) | 11,198  (0 – 44,143) |
| SD2 | 0 (0 – 0) | not observed | 0 (0 – 0) | 0 (0 – 0) | 0 (0 – 0) |
| SD3 | 68 (0 – 971) | not observed | 53 (0 – 857) | 718  (0 – 4,084) | 239  (0 – 2,349) |
| SD4 | 434  (0 – 2,495) | not observed | 168  (0 – 1,978) | 4,084  (0 – 3,517) | 473  (0 – 3,940) |
| **Karasik Mount** | | | | | |
| SD1 | 14,977  (0 – 40,699) | 12,488  (6,651 – 15,731) | 3,157  (0 – 18,823) | 44,721  (11,970 – 89,963) | 28,158  (2,610 – 64,262) |
| SD2 | 0 (0 – 0) | 0 (0 – 0) | 0 (0 – 0) | 67  (0 – 1,310) | 64  (0 – 1,480) |
| SD3 | 312  (0 – 2,906) | 0 (0 – 0) | 589  (0 – 2,883) | 811  (0 – 4,731) | 0  (400 – 3,862) |
| SD4 | 1,256  (0 – 8,405) | 0 (0 – 0) | 194  (0 – 1,277) | 784  (0 – 6,218) | 1,896  (0 – 11,199) |
| **Northern Mount** | | | | | |
| SD1 | 16,742  (0 – 53,841) | 4,169  (192 – 8,418) | 75 (0 – 733) | 38,627  (9,776 – 92,838) | 4,638  (0 – 59,836) |
| SD2 | 0 (0 – 0) | 0 (0 – 0) | 0 (0 – 0) | 55 (0 – 792) | 0 (0 – 0) |
| SD3 | 179  (0 – 1,135) | 99 (0 – 281) | 0 (0 – 0) | 1,066  (0 – 5,887) | 122  (0 – 1,695) |
| SD4 | 2,031  (0 – 18,005) | 0 (0 – 0) | 41 (0 – 398) | 585  (0 – 5,087) | 212  (0 – 3,605) |

**Table S6.** Relative abundance (%) of phospholipid derived fatty acids (PLFAs) from specimens collected at the Karasik Seamount. Data are reported as mean ± standard deviation.

| **PLFAs** | **Biomarker*** | **Starfish** (n = 1) | **Microbial mat covering sponges in decay stage SD2** (n = 2) |
| --- | --- | --- | --- |
| C16:0 | palmitic acid | 4.69 | 5.65 |
| C14:0 | general | 3.35 | 1.11 |
| C15:0 | general | 0.41 | 0.37 |
| C18:0 | general | 3.76 | 3.80 |
| C20:0 | general | 0.47 |  |
| C23:0 | general | 0.74 | 0.24 |
| *i*C15:0 | bacteria | 1.56 | 3.71 |
| *ai*C15:0 | bacteria | 0.90 | 1.94 |
| *i*C17:0 | bacteria | 0.30 | 0.96 |
| *ai*C17:0 | bacteria | 0.62 | 0.34 |
| Me-C14:0 | bacteria | 0.32 | 1.64 |
| Me-C15:0 | bacteria | 0.73 | 1.56 |
| 10-Me-C17:0 | bacteria | 2.76 | 1.56 |
| 8/9/10/11-MeC16:0 | bacteria | 3.63 | 10.9 |
| 9/10/11-MeC18 | bacteria | 4.22 | 21.0 |
| *(a)i*16:1ω | bacteria | 0.33 | 0.37 |
| C16:1ω(5/7/9) | bacteria | 3.29 | 11.0 |
| iC17:1ω7 | bacteria | 0.38 | 1.27 |
| C18:1ω(9/7/9t) | bacteria | 7.50 |  |
| C18:1ω9c | bacteria | 3.25 | 0.80 |
| *i*C19:1ω12 | bacteria | 0.52 | 2.53 |
| Cy-C17:0 | bacteria | 0.15 |  |
| Cy-C19:0 | bacteria | 1.41 |  |
| C20:3ω6 | algae | 0.90 |  |
| C20:4ω6/C20:5ω3 | algae | 0.86 |  |
| C22:6ω3 | algae | 0.54 |  |
| C18:2ω6c | algae | 2.04 |  |
| C18:3ω6 | algae | 11.2 |  |
| C20:2ω_1 | algae | 4.27 |  |
| C20:2ω_2 | algae | 2.26 |  |
| C22:2ω | - | 0.54 | 0.86 |
| C22:1ω? |  | 0.25 |  |
| C24:2ω? | sponge | 0.27 | 0.60 |
| C24:1ω9c |  | 0.11 |  |
| C25:2ω? | sponge | 0.60 | 6.55 |
| Me24:2ω | sponge | 1.57 | 5.37 |
| 26:ω2? | sponge | 0.11 |  |
| 26:2ω | sponge | 0.12 |  |
| 28:2ω?_1 | sponge |  | 1.60 |
| 28:3ω | sponge | 0.30 |  |
| 30:3ω?/32:1ω | sponge |  | 0.14 |
| 20:1ω9c |  | 10.7 |  |
| 20:1ω5 |  | 6.33 |  |
| 22:2ω6 |  | 0.33 |  |
| 22:1ω9c |  | 0.62 |  |

*Biomarker identified as described in (de Kluijver et al., 2021).

**Table S7.** Ocean Floor Observation and Bathymetry System (OFOBS) transects. For each seamount, photos of one to two OFOBS transects were analyzed.

| **OFOBS transect** | **Start of bottom transect; depth** | **End of bottom transect; depth** | **Analyzed images** | **Seafloor area imaged (m^2^)** | **Seamount** |
| --- | --- | --- | --- | --- | --- |
| PS101/100-1 | 86°49.39' N, 61°57.84' E; 988 m | 86°47.93' N, 61°52.11' E; 722 m | 324 | 1955 | Central Mount |
| PS101/169-1 | 86°45.67' N, 61°51.86' E; 878 m | 86° 48.32' N, 61°47.41' E; 838 m | 639 | 5301 | Central Mount |
| PS101/089-1 | 86°42.18' N, 61°13.05' E; 567 m | 86°44.02' N, 61°30.01' E; 608 m | 824 | 5359 | Karasik Seamount |
| PS101/120-1 | 86°51.93' N, 61°19.55' E; 967 m | 86°51.13' N, 62°00.19' E; 2117 m | 312 | 5076 | Northern Mount |


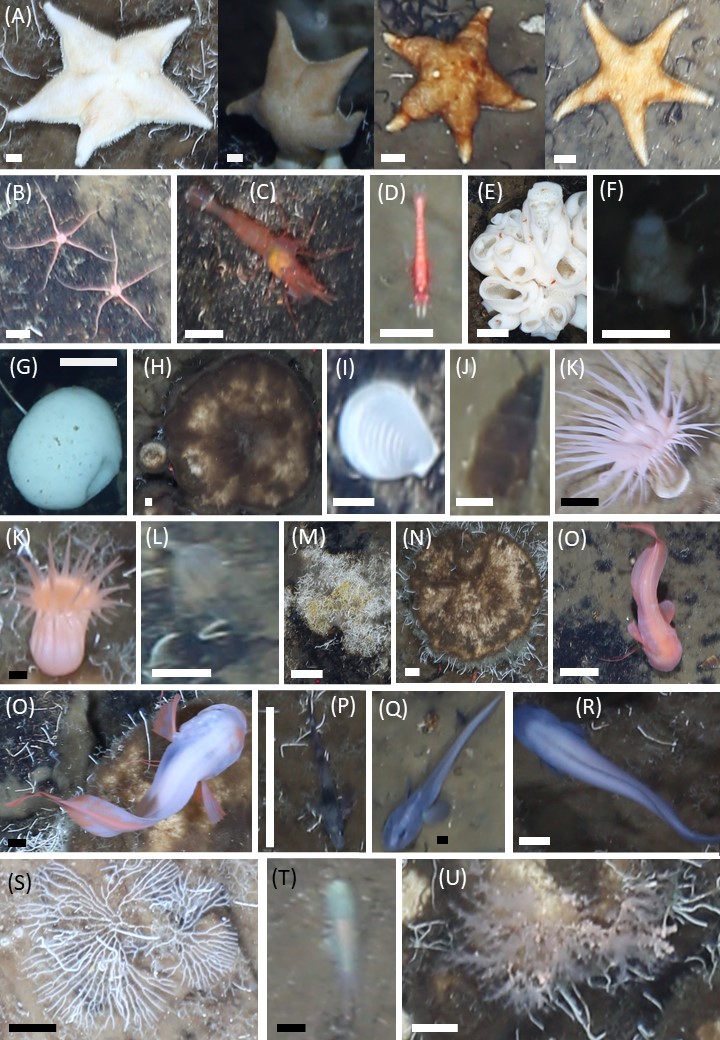


**Figure S1.** Images of all megafauna observed at the Central Mount, the Karasik Seamount, and the Northern Mount. (A) *Tylaster willei* sp. inc., (B) *Ophiostriatus striatus* sp. inc., (C) *Bythocaris* sp. indet., (D) *Neobirsteiniamysis inermis* sp. inc., (E) *Schaudinnia rosea* sp. inc., (F) *Schaudinnia rosea* sp. inc. juv., (G) *Hyalonema* sp. indet., (H) Tetractinellida gen. indet., (I) *Hyalopecten frigidus* sp. inc., (J) Buccinidae fam. indet., (K) Hormathiidae gen. indet., (L) Edwardsiidae gen. indet., (M) Serpulidae gen. indet. and Siboglinidae gen. indet., (N) *Apomatus globifer* sp. inc./ *Hyalopomatus claparedii* sp. inc., (O) *Rhodichthys regina* inc., (P) Gadidae fam. indet., (Q) *Lycodes* sp. indet., (R) Liparidae fam. indet., (S) Cyclostomatida fam. indet., (T) Macellicephalinae gen. indet., (U) Nephtheidae gen. indet.

The black/ white bar represents 1 cm length, except for (H), (L), and (S) where the bar represents 2.5 cm length and 5 cm length for (D), (F), (N), and (R).


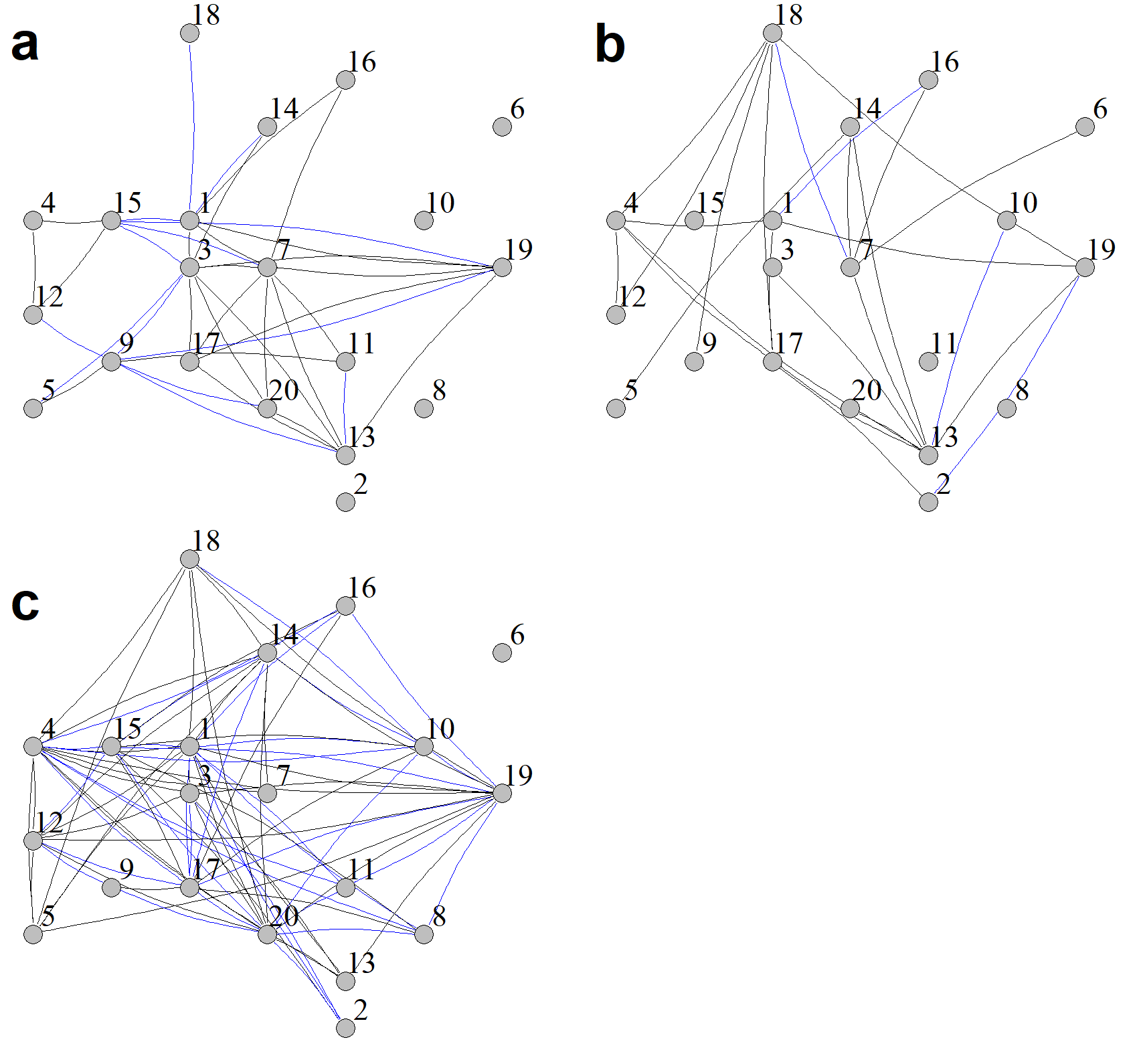


**Figure S2.** Co-occurrence network plots of the (a) Central Mount, (b) Karasik Seamount, and (c) Northern Mount faunal community. The black lines show two taxa co-occurring at a higher frequency than expected by chance (*i.e.,* *p_gt_* < 0.05), whereas the blue lines correspond to two taxa co-occurring at a lower frequency than expected by chance (*i.e.,* *p_lt_* < 0.05).

The numbers correspond to the following taxa: 1 *Apomatus globifer* sp. inc./ *Hyalopomatus* *claparedii* sp. inc., 2 Buccinidae fam. indet., 3 *Bythocaris* sp. indet., 4 Cyclostomatida fam. indet., 5 Edwardsiidae gen. indet., 6 Gadidae fam. indet., 7 Hormathiidae gen. indet., 8 *Hyalonema* sp. indet., 9 *Hyalopecten frigidus* sp. inc., 10 Liparidae fam. indet., 11 *Lycodes* sp. indet., 12 Macellicephalinae gen. indet., 13 *Neobirsteiniamysis inermis* sp. inc., 14 Nephtheidae gen. indet., 15 *Ophiostriatus striatus* sp. inc., 16 *Rhodichthys regina* inc., 17 *Schaudinnia rosea* sp. inc., 18 Serpulidae gen. indet. and Siboglinidae gen. indet., 19 Tetractinellida gen. indet., 20 *Tylaster willei* sp. inc.
